# Mapping the future: The current landscape and future directions of evidence-based practice in Saudi radiology departments

**DOI:** 10.1371/journal.pone.0314332

**Published:** 2025-02-04

**Authors:** Walaa Alsharif, Faisal Alrehily, Fahad H. Alhazmi, Awadia Greeballah, Abdulaziz A. Qurashi, Shrooq Aldahery, Adnan Alahmadi, Amirah Alsaedi, Sultan Alshoabi, Khalid M. Alshamrani, Awatif M. Omer, Raghad Aljuhani

**Affiliations:** 1 Department of Diagnostic Radiology, College of Applied Medical Sciences, Taibah University, Madinah, Saudi Arabia; 2 Applied Radiologic Technology, College of Applied Medical Science, University of Jeddah, Jeddah, Saudi Arabia; 3 Radiologic Sciences Department, Faculty of Applied Medical Sciences, King Abdulaziz University, Jeddah, Saudi Arabia; 4 College of Applied Medical Sciences, King Saud bin Abdulaziz University for Health Sciences, Jeddah, Saudi Arabia; 5 King Abdullah International Medical Research Center, Jeddah, Saudi Arabia; 6 Ministry of the National Guard—Health Affairs, Jeddah, Saudi Arabia; University of Hafr Al-Batin, SAUDI ARABIA

## Abstract

**Purpose:**

To examine the current application of Evidence-Based Practice (EBP) among radiology professionals, including radiologists and radiographers, in Saudi Arabia and to identify challenges in order to propose suitable future improvement strategies if it is required.

**Method:**

A mixed-method design was used in this study. A survey consisting of 23 questions addressing research activities and EBP within radiology departments was sent to radiology personnel. The sample size of the quantitative phase of the study was determined using a formula specific for an infinite or unknown population. The formula used was n = P(1-P)Z^2^/d^2^, which resulted in a required sample size of 384 participants. A total of 345 participants; however, 45 did not fully complete the questionnaire and were therefore excluded. The data were analyzed using SPSS version 27. Inferential statistics, including non-parametric tests such as the Mann-Whitney U Test and the Kruskal-Wallis Test, were used to assess the influence of demographic factors on perceptions and challenges related to the adoption of evidence-based practice (EBP) in research within Saudi Arabia. Additionally, 20 semi-structured interviews were conducted with radiology personnel across the country. The sampling technique for the qualitative phase of the study was guided by the study’s objectives and the unique characteristics of the research group. The participants were purposively sampled in order to include radiologists and radiographers who work in different types of hospitals (public, semi-public, private) in Saudi Arabia. Responses from the interviews were coded, and key themes were identified following Miles and Huberman’s framework.

**Results:**

The findings revealed a positive attitude towards research and EBP among Saudi radiology personnel. Over half of the participants (74.3%) strongly agreed that they understood and were familiar with EBP. They also felt confident in their ability to conduct scientific research in radiology (Mean = 4.27) and believed that they should actively initiate projects (Mean = 4.10). Radiologists reported a higher level of agreement compared to radiographers regarding their familiarity with EBP and their ability to critically evaluate the quality of research (P-value = <0.05). However, participants indicated lower level of agreement about their ability to develop their current practice based on EBP and engage in discussions with colleagues about research evidence. Key challenges identified include a lack of training, insufficient support and limited autonomy, which may hinder EBP implementation.

**Conclusion:**

This study underscores the need for comprehensive education, ongoing training and a supportive organisational culture to enhance EBP adaption.

## Introduction

Ensuring the quality of care delivered to patients has become a top priority for all healthcare providers [1]. Therefore, involving tasks such as Evidence-Based Practice (EBP) is critical to meet healthcare demand and quality benchmarks [2]. EBP is the practical underpinning for clinical practice by healthcare professionals globally [3, 4]. It enables healthcare personnel to gain knowledge from valid and relevant research evidence, combined with clinical expertise, to make decisions regarding patient care and achieve optimal outcomes [4, 5]. EBP is a cyclical process that starts with a clear clinical question, involves conducting deep literature searches, critically appraises the available evidence and evaluates the applicability of outcomes in practice [6, 7].

When healthcare professionals, such as radiology experts, deliver healthcare services without considering the current existing research evidence, they may overlook optimal decisions that could benefit patients, potentially leading to significant harm instead [8]. A thorough review of studies from 2012 to 2017, focusing on healthcare professionals’ capabilities in EBP revealed that many reported having a good grasp of EBP knowledge, skills and attitudes. However, this may not necessarily translate to using EBP in their current practice, highlighting the disconnection between their knowledge about EBP and the importance of applying it in real-world healthcare settings [9]. A recent survey conducted by Ehrenbrusthoff et al. across Germany found a positive attitude towards EBP among participants, indicating a favourable view on implementing EBP in their professional activities [10]. Further study showed that although nurse-midwives are involved to a certain degree with EBP, there are still obstacles (e.g. research skills, skills to apply EBP) to consider EBP as a regular part of their daily work routines [11].

Despite the positive attitude of healthcare personnel towards EBP, it seems that there is a deficiency in its implementation of this method, and this practice is still not well-established among healthcare professionals, including radiology professionals [12]. Studies showed that radiologists understand the significance of EBP in making clinical decisions and caring for patients. Yet, they face hurdles in fully integrating and utilizing EBP due to difficulties in accessing evidence, lack of education and training, and a lack of practical tools for evaluating the evidence, which hampers their ability to effectively use EBP [13, 14]. Ramazan et al. stated that radiographers generate and utilise evidence in their work, although EBP is not yet routinely incorporated as established practice [15]. An additional study revealed that although radiographers have a basic understanding of EBP, and they consider its value, many of them feel unconfident in applying EBP in their work. The biggest challenge for this is time constraint [16].

Integrating EBP in radiology practice is a process that requires changes in radiology professionals’ perceptions and attitudes and creating a supportive research culture at organisational level [5, 17, 18]. Radiology experts’ perceptions of their organisational culture may directly impact their engagement in research, their understanding of the value of research evidence and their ability to embrace EBP within their current practice [3, 19, 20]. Several studies have investigated the barriers of implementing EBP and research in healthcare, including clinical radiography. However, there are limited studies in the literature that focus specifically on EBP in radiology, and those that did typically examine the views of either radiologists or radiographers separately. To the best of the researchers’ knowledge, this is the first study to investigate the current application of EBP among radiology professionals, including radiologists and radiographers, in Saudi Arabia and to identify challenges intending to propose suitable future improvement strategies if it is required.

## Materials and method

### Ethical consideration

This study was approved by the Institutional Review Board in the College of Applied Medical Science at Taibah University (Reference Number: 2021/109/301). All research participants retained their right to choose whether to participate in this study, and written informed consent was obtained from those who agreed to participate.

### Study design and data collection

**Fig 1:**
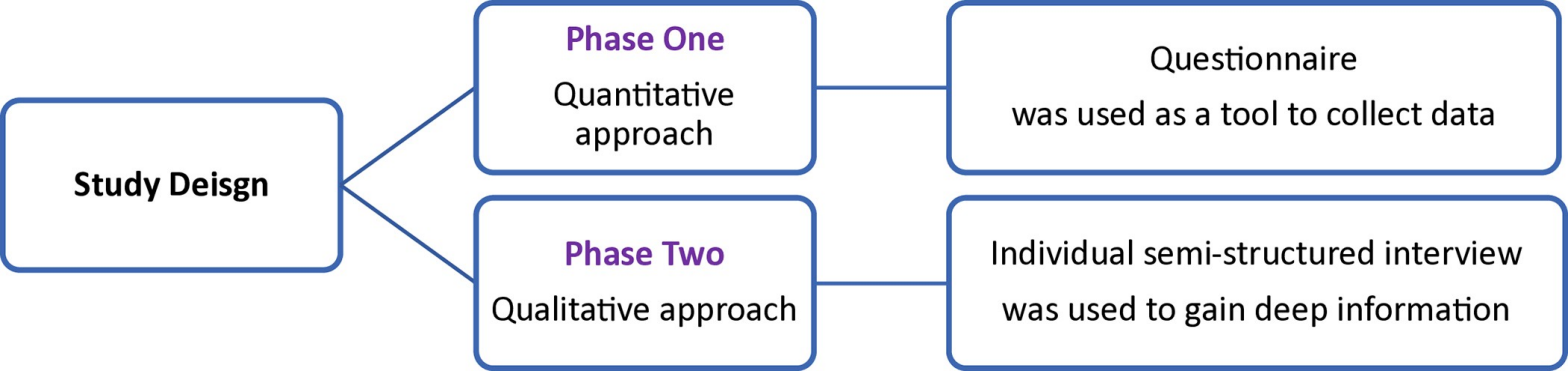
Study design.

A mixed-method approach was used to achieve the study aim (Fig 1). Radiology personnel (radiologists and radiographers) across the Kingdom of Saudi Arabia were targeted and included in this study, and other professionals were excluded. The results of phase (1) informed the development and design of the tool for phase (2). The sample size for phase (1) (n) was calculated using the formula for sample size with an infinite (unknown) population for qualitative research: (n = P (1-P) Z2/ d2) with a margin of error (d) of 0.05, Z value of 1.96 (for a 95% confidence interval), and a population proportion of 0.5. This calculation resulted in a required sample size of 384.

During the initial phase, an online questionnaire was sent to the study participants across various professional social media platforms from December 2023 to February 2024. The questionnaire was developed in compliance with previous studies [8, 13, 15, 21] and was amended and designed to ascertain its relevance to the study participants. A pilot study was conducted to validate the adjusted questionnaire prior to starting the main study with four academic lecturers and 20 radiology professionals to ensure that all survey questions were clearly presented and easy to understand. A few amendments were made to the questionnaire according to the pilot responses received. All study participants were informed about the study aims, and confidentiality was granted. An informed consent agreement was obtained at the beginning of the questionnaire. The questionnaire was available to the participants in two versions (Arabic and English).

Demographic characteristics about study participants, including place of work, years of experience, qualifications and job description, was collected. The questionnaire contains 23 items covering four areas: **(a)** research activities in radiology, **(b)** understanding and proficiency in EBP within radiology, **(c)** challenges in adapting EBP within radiology departments, **(d)** perspective about students’ research and EBP, which explored the current practice of EBP in radiology departments across the country. A five-point Likert scale was used to indicate the level of agreement among the participants, (1 = strongly disagree (SDA), 2 = disagree (DA), 3 = neutral (NE), 4 = agree (AG), 5 = strongly agree (SAG)).

In phase (2) of the study, a qualitative method was employed using semi-structured interviews.

This method was deemed suitable for obtaining a deeper comprehension of the responses provided in the questionnaires.

Semi-structured interviews (n = 20) were individually conducted using purposive sampling of Saudi diagnostic radiology professionals. The researchers continued data collection until no new data emerged (i.e., until saturation was reached). Sampling in qualitative research is determined by the purpose of the study and focuses more on individual voices rather than on statistical representativeness as outlined in Miles and Huberman farmwork. Therefore, the selection of the sampling technique in this phase of the study was guided by the study’s objectives and the distinctive characteristics of the research group. The participants in this phase were purposively sampled in order to include radiologists and radiographers who work in different types of hospitals (public, semi-public, private) in Saudi Arabia. The inclusion criteria for the study were that participants who work in radiology departments needed to be either radiologists or radiographers. To include staff who were involved in the service delivery, they were invited to participate in this phase of the study, which involved them being interviewed by the researcher in the diagnostic imaging department at their place of work. The interview questions were formulated to achieve a more profound understanding of the themes identified in the questionnaire.

**Fig 2:**
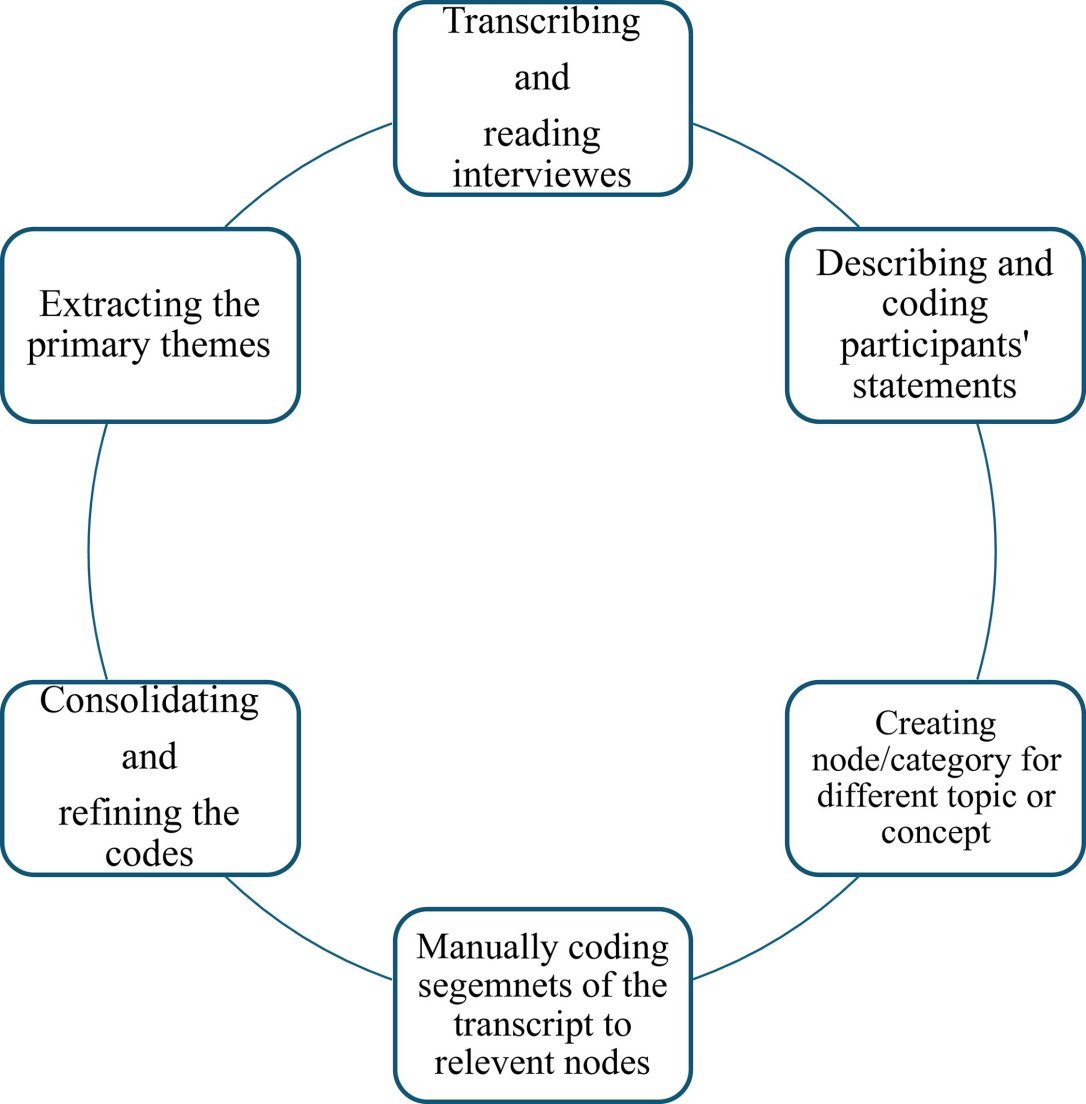
Coding process for qualitative data.

Given the challenges associated with travelling across the country to conduct participant interviews, the researchers opted to conduct virtual semi-structured interviews via video calls with each participant. The interview sessions were conducted in English and were scheduled for 40-minute intervals. Permission was sought to digitally record the interview responses. Subsequently, the researchers manually transcribed the recorded interviews. The transcripts were de-identified, and codes were assigned to quotes to safeguard the anonymity of the participants. Following the transcription, the data underwent a coding process by the researcher (Fig 2).

### Data analysis

Likert scale data were analysed using *IBM Software Platform Offers Advanced Statistical Analysis* (SPSS) version 27, and a P value of <0.05 was considered statistically significant. Frequency and percentage distributions were used to demonstrate the categorical values, including demographic characteristics.

The study respondents’ perspectives on research activities and the current practice of EBP within radiology departments were assessed through a set of 23 questions across four domains. Responses were rated on a five-point Likert scale. Means and standard deviations were calculated for each question within the domains (Mean ± SD), and a weighted average for each domain was computed. A normality test was performed on the continuous data. The Kolmogorov-Smirnov and Shapiro-Wilk tests both indicated that the data was not normally distributed, with a p-value of <0.001 for the mean score across all four domains. Histogram analysis further revealed deviations from the bell curve, confirming non-normality of the data. Consequently, non-parametric tests were employed to assess the correlations. The Independent-Samples Mann-Whitney U Test was used for two groups of demographic factors, while the Kruskal-Wallis Test was applied for three or more independent demographic groups.

Inductive thematic analysis was performed on the interviews using *Nvivo* software version 14. The interview responses were coded, and common themes were identified in alignment with the philosophical principles of Miles and Huberman (Fig 2).

## Results

### Quantitative findings

Study findings were analysed to explore the current status of the EBP implementation in radiology departments across Saudi Arabia. The internal consistency for questionnaire questions was satisfactory, with Cronbach’s alpha coefficients (α) demonstrating acceptable reliability (α > 0.70). Of the potential participants, 345 respondents agreed to participate, yielding a response rate of 89.9%. However, 45 respondents did not fully complete the questionnaire and were subsequently excluded from the analysis. Table 1 indicates the demographic characteristics of the study participants.

**Table 1:** Demographic characteristics of the study sample.

Demographic		N (%)
Gender	Male	129(43)
	Female	171(57)
Job description	Radiologists	65(21.7)
	Radiographers	235 (78.3)
Qualification	Diploma	30(10)
	BSc	188(62.7)
	MSc	8(2.7)
	PhD	9(3)
	MD	65(21.7)
Hospital types	Public hospital	124(41.3)
	Semi-public hospital	87(29)
	Academic hospital	35(11.7)
	Private hospital	54(18)
Years of experience	1–5	155(51.7)
	6–10	73(24.3)
	>10	72(24)
Participation in a research activity	Yes	193(64.3)
	No	107(35.7)
Familiarity with research strategy in workplaces	Yes	171 (57)
	No	129 (43)
Total	300	

Table 2 displays the current perspectives and attitudes of radiology department personnel regarding engagement in research activities and the implementation of EBP. While inquiring regarding the research activities among study participants in radiology, the respondents answered eight questions using a five-point Likert scale. The overall mean score was found to be 4.07, indicating a high perception concerning research activities in radiology. The majority of the study participants expressed strong agreement regarding the role of scientific research in advancing the radiology profession and informing clinical decision-making. More than half of the participants expressed confidence in their abilities to conduct scientific research in radiology and believed that they should play an active role in initiating projects. The findings revealed diverse opinions and attitudes regarding who should initiate and lead research activities in radiology. However, 69% of the participants emphasised the necessity of cooperation between academic institutions and healthcare organisations in conducting research in radiology.

**Table 2:** Perspective concerning research activities and EBP within radiology departments.

	SDA	DA	NE	AG	SAG	Mean	σ	95%(CI)	Decision
Domain 1—Research activities in radiology	N (%)								
**1.1** Research is required to promote the radiology profession about research in radiology	0 (0.0%)	8 (2.7%)	11 (3.7%)	87 (29%)	194 (64.7%)	4.56	0.69	4.48–4.64	High
**1.2** Clinical decision in radiographic practice should be based on research evidence	0 (0.0%)	3 (1.0%)	14 (4.7%)	97 (32.3%)	186 (62%)	4.55	0.63	4.48–4.63	High
**1.3** You as a radiographer/radiologist is competent to conduct research in radiology field	0 (0.0%)	11 (3.7%)	45 (15%)	96 (32%)	148 (49.3%)	4.27	0.84	4.17–4.37	High
**1.4** You as a radiographer/Radiologist should be initiators of radiographic research projects	0 (0%)	12 (4%)	69 (23%)	97 (32.3%)	122 (40.7%)	4.10	0.88	4.0–4.20	High
**1.5** You as a radiographer/radiologist should oversee radiographic research projects	2 (0.7%)	39 (13%)	73 (24.3%)	65 (21.7%)	121 (40.3%)	3.88	1.10	3.75–4.01	Low
**1.6** Radiographic research projects should be initiated and led by healthcare institutions (e.g. MOH) only	10 (3.3%)	91 (30.3%)	51 (17%)	57 (19%)	91 (30.3%)	3.43	1.29	3.28–3.57	Low
**1.7** Radiographic research projects should be initiated and led by academic institutions only	26 (8.7%)	96 (32%)	58 (19.3%)	49 (16.3%)	71 (23.7%)	3.14	1.32	2.99–3.29	Low
**1.8** Cooperation between educational and academic and healthcare institutions in conducting radiography research is important	0 (0.0%)	2 (0.7)	20 (6.7%)	70 (23.3%)	208 (69.3%)	4.61	0.64	4.54–4.69	High
**Domain 2—Understanding and proficiency in EBP within radiology departments**									
**2.1** I am familiar with evidence-based research in my profession or/and specialty	3 (1.0%)	19 (6.3%)	55 (18.3%)	153 (51%)	70 (23.3%)	3.89	0.86	3.79–3.99	High
**2.2** I evaluate critically the quality of research to provide evidence-based practice in my place of work	6 (2%)	22 (7.3%)	69 (23%)	151 (50.3%)	52 (17.3%)	3.74	0.90	3.63–3.84	High
**2.3** I develop my current practice on the grounds of evidence-based research	23 (7.7%)	18 (6%)	66 (22%)	128 (42.7%)	65 (21.7%)	3.65	1.11	3.52–3.77	Low
**2.4** I discuss research evidence with my colleagues(radiographers/radiologists)	20 (6.7%)	29 (9.7%)	39 (13%)	139 (46.3%)	73 (24.3%)	3.71	1.13	3.59–3.85	Low
**2.5** I discuss research evidence with other professionals such as radiologists/radiographers	30 (10%)	17 (5.7%)	56 (18.7%)	126 (42%)	71 (23.7%)	3.64	1.19	3.50–3.77	Low
**Domain 3—Challenges in adopting EBP within radiology debarments**									
**3.1** Lack of knowledge and research skills (e.g., discuss or evaluate the research)	1 (0.3%)	12 (4.0%)	60 (20.0%)	141 (51%)	86 (28.7%)	4.0	0.82	3.90–4.09	High
**3.2** Lack of resources (e.g., access to research)	12 (4%)	27 (9%)	45 (15%)	136 (45.3%)	80 (26.7%)	3.82	1.05	3.70–3.94	Low
**3.3** Lack of teamwork	4 (1.3%)	48 (16%)	53 (17.7%)	124 (41.3%)	71 (23.7%)	3.70	1.04	3.58–3.82	Low
**3.4** Lack of autonomy/authority to apply change	0 (0.0%)	23 (7.7%)	61 (20.3%)	131 (43.7%)	85 (28.3%)	3.93	0.88	3.83–4.03	High
**3.5** Lack of support	1 (0.3%)	19 (6.3%)	52 (17.3%)	127 (42.3%)	101 (33. 7%)	4.03	0.89	3.93–4.13	High
**3.6** Insufficient time	14 (4.7%)	42 (14%)	52 (17.3%)	93 (31%)	99 (33%)	3.74	1.19	3.60–3.87	Low
**Domain 4—Student research and EBP within radiology departments**									
**4.1** I discuss research evidence with students in their clinical practice	17 (5.7%)	25 (8.3%)	48 (16%)	92 (30.7%)	118 (39.3%)	3.90	1.17	3.76–4.03	Low
**4.2** I encourage students to search evidence-based practice	0 (0.0%)	6 (2%)	33 (11%)	101 (33.7%)	160 (53.3%)	4.38	0.76	4.30–4.47	High
**4.3** I am willing to participate in research with students to support evidence-based practice	0 (0.0%)	12 (4%)	17 (5.7%)	108 (36%)	163 (54.3%)	4.41	0.77	4.32–4.49	High
**4.5** I find graduation projects of students is an opportunity to support evidence-based practice	1 (0.3%)	16 (5.3%)	33 (11%)	80 (26.7%)	170 (56.7%)	4.34	0.89	4.24–4.44	High

In terms of understanding and proficiency in EBP within radiology departments, respondents answered five questions rated on a five-point Likert scale. The overall mean score was found to be 3.73, indicating moderate familiarity with EBP in radiology. Most respondents confirmed their familiarity with EPB in their profession, with a mean score of 3.89, where 74.3% either strongly agreed or agreed. Additionally, 67.6% either strongly agreed or agreed they agreed that they critically evaluate the quality of research to provide EBP in their workplace. In contrast, below-average mean scores were found for developing their current practice based on evidence-based research (Mean = 3.65), discussing research evidence with their colleagues (Mean = 3.71) and engaging in discussions with other professionals (Mean = 3.64).

The barriers to implement EBP in the radiology department were assessed by asking participants six questions. The mean score was found to be 3.78, indicating a stronger perception of the item as a barrier to EBP implementation. The major barriers identified as obstacles to the implementation of EBP in radiology departments were a lack of knowledge and research skills (79.7%, Mean = 4), a lack of autonomy/authority to apply changes (72%, Mean = 3.9), and a lack of support (76%, Mean = 4).

The viewpoints of radiographers and radiologists concerning student projects and EBP were assessed through four questions. The overall mean score was 4.26, indicating a high perception among the study participants regarding students’ projects and EBP. The study participants showed a strong willingness to engage in research with students to promote EBP (90%, Mean = 4.41) and viewed students’ graduation projects as opportunities to advance EBP (83%, Mean = 4.34). Most of the respondents strongly agreed or agreed with encouraging students to pursue EBP (87%, Mean = 4.3).

**Fig 3:**
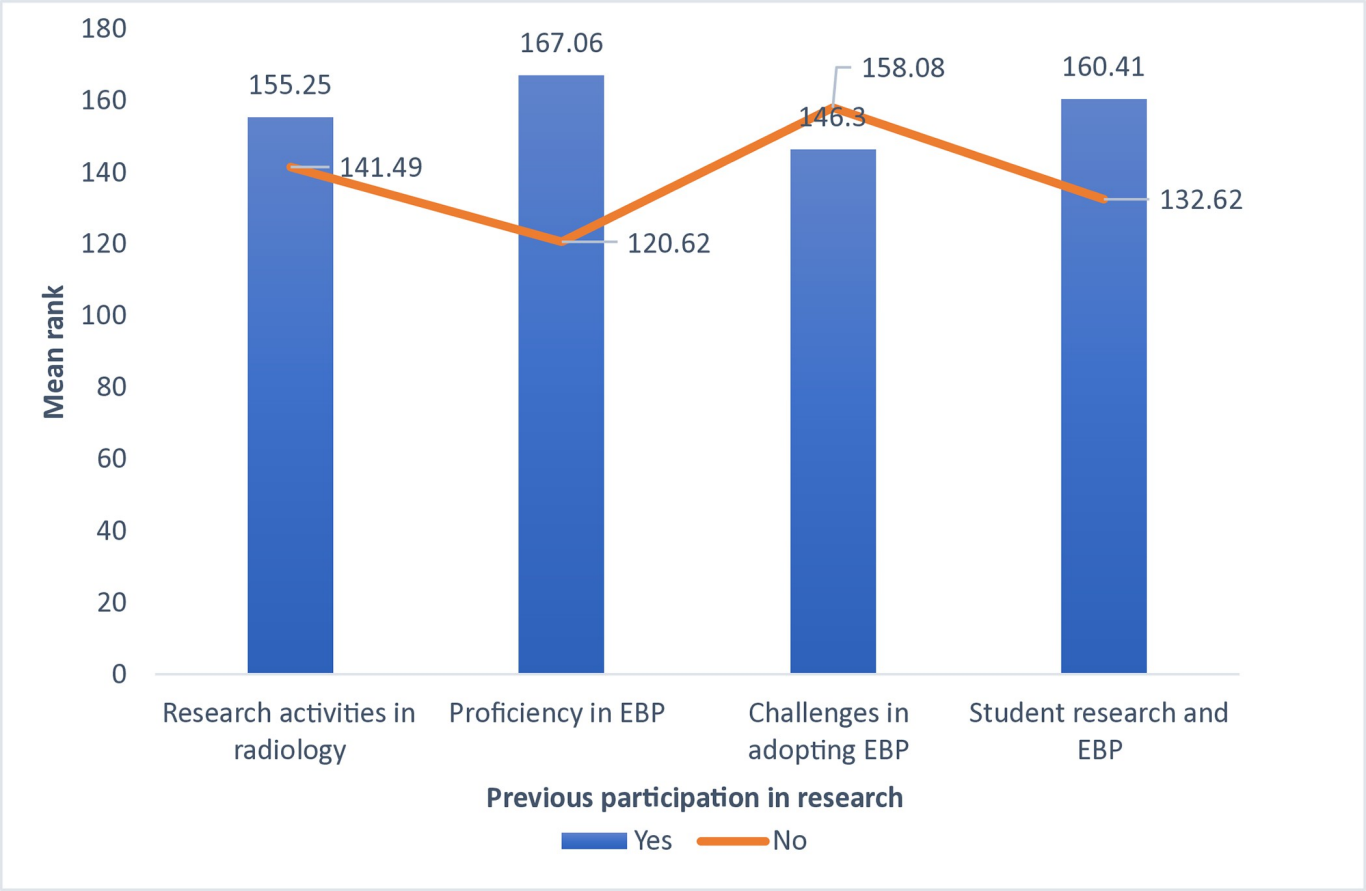
Comparison of mean rank scores for research activities and EBP proficiency based on previous participation in research.

**Fig 4:**
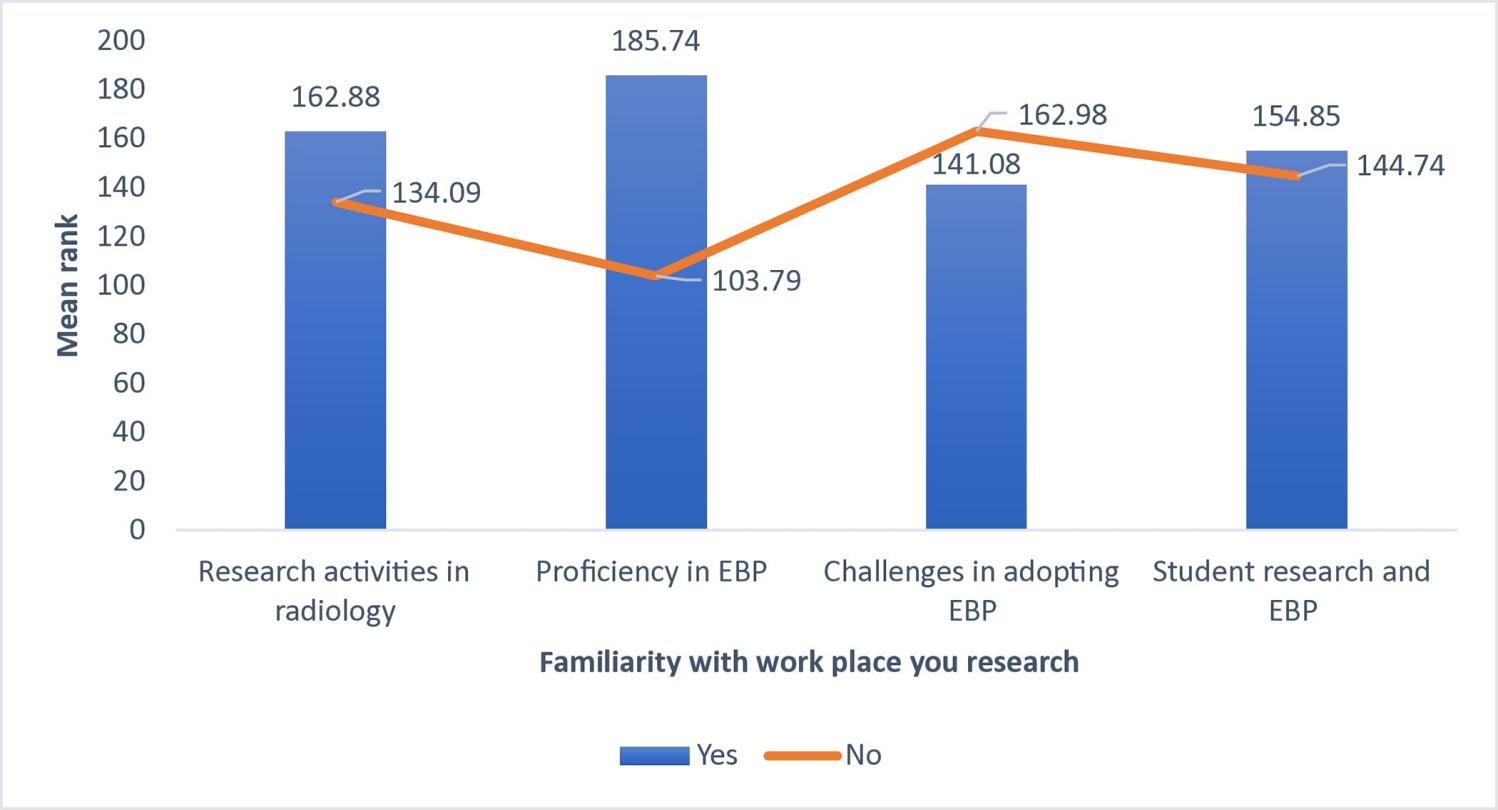
Comparison of mean rank scores for research activities and EBP proficiency based on familiarity with workplace research.

The study findings demonstrated a significant difference between previous participation in a research activity and the level of agreement concerning research and the current practice of EBP within radiology departments (p<0.05). The participants who had previously taken part in research had a higher mean rank in research and current practice of EBP compared to those who had not participated (p<0.05) (Fig 3). A significant difference was also found in mean rank between individuals who were familiar with research strategy in their workplaces and their level of agreement concerning research and current practice of EBP within the radiology department (p<0.05). Those who were familiar with research strategy in their workplaces expressed the highest mean ranks compared to others (Fig 4).

No significant difference was found across all domains based on participants’ gender and job description. However, radiologists expressed higher level of agreement than radiographers concerning research activities and EBP in radiology (Table 3). The study results showed a significant difference between participants’ responses based on their workplace. Those who work in academic hospitals expressed higher agreement concerning research activities and EBP in radiology compared to those who work in other settings. Regarding challenges in adapting EBP within radiology debarments, participants working in private hospitals exhibited higher mean rankings, followed by those who work in academic hospitals (Table 3).

**Table 3:** Comparison for four domains according to the sociodemographic variables: (Gender, job description, hospital types).

Domains		Gender			Job description			Hospital types					
		Male	Female	P-vale	Radiologists	Radiographers	P-vale	Public	Semi-public	Academic		private	P-value
		Mean Rank			Mean Rank			Mean Rank					
D-1	1.1	158.36	144.57	100015 (0.104)	142.03	181.13	9628.5 (<0.001)	130.47	164.74		187.44	149.61	21713 (<0.001)
	1.2	138.59	159.49	12566 (0.015)	142.8	178.35	9447.5 (<0.001)	135.25	163.01		179.20	146.77	13157 (0.004)
	1.3	147.59	152.70	11405 (0.583)	142.82	178.27	9442.5 (0.002)	145.46	132.79		188.96	165.67	14901 (0.002)
	1.4	165.63	139.08	90077.5 (0.005)	143.79	174.1	9171.5 (0.008)	135.15	138.87		212.86	164.06	28022 (<0.001)
	1.5	163.70	140.54	9326.5 (0.016)	147.34	161.49	8381.0 (0.207)	158.44	138.77		201.29	118.25	24338 (<0.001)
	1.6	152.78	144.77	10734 (0.681)	163.26	104.37	4639.0 (<0.001)	150.64	143.22		117.66	183.09	14203 (0.003)
	17	138.66	159.43	12557 (0.034)	155.91	130.92	6365.0 (0.034)	142.12	143.12		131.53	193.92	18037 (<0.001)
	1.8	160.80	142.73	9700.5 (0.027)	145.68	168.01	8775.5 (0.023)	143.46	143.79		192.53	150.24	14615 (0.002)
	**Overall**	158.36	144.57	10015 (0.333)	148.03	159.42	8217.0 (0.347)	140.10	139.26		197.76	165.76	12940 (0.005)

D-2	2.1	145.27	154.45	11704.5 (0.324)	142.76	178.48	9456.0 (<0.001)	157.06	140.60		155.09	148.42	2322 (0.508)
	2.2	148.85	151.75	11242.5 (0.757)	145.45	168.76	8.824.5 (0.038)	148.08	137.75		179.73	157.66	7390 (0.060)
	2.3	172.62	133.81	8176.0 (<0.001*)	150.40	150.85	7660.5 (0.969)	163.4	145.29		167.46	118.29	13143 (0.004)
	2.4	169.81	135.93	8538.0 (<0.001*)	150.24	151.45	7699.5 (0.915)	159.11	145.86		165.51	128.46	6802 (0.078)
	2.5	161.17	142.45	9652.5 (0.052)	151.63	146.42	7372 (0.652)	161.60	156.84		155.70	111.42	15010 (0.002)
	**Overall**	165.61	139.10	9080.0 (0.008*)	145.61	168.17	8786 (0.061)	162.51	151.66		175.47	104.87	20639 (<0.001)

D-3	3.1	162.32	141.58	9505.0 (0.028*)	150.73	149.68	7584.5 (0.927)	164.70	111.27		136.66	190.06	34438 (<0.001)
	3.2	14.55	158.01	12313.5 (0.066)	150.20	151.58	7708.0 (0.904)	160.90	120.63		159.64	168.81	16851 (0.001)
	3.3	162.00	141.82	9546 (0.036*)	151.39	147.28	7428.0 (0.722)	145.38	121.76		176.53	191.70	27928 (<0.001)
	3.4	153.53	148.21	10638 (0.576)	153.65	139.11	6897.0 (0.204)	145.02	138.83		157.54	177.33	8438 (0.038)
	3.5	155.36	146.83	10402.0 (0.369)	154.06	137.62	6800.5 (0.150)	150.83	128.67		149.73	185.42	16197 (0.001)
	3.6	151.77	149.55	10866.0 (0.819)	153.47	139.75	6939.0 (0.241)	153.44	170.70		155.67	107.87	19458 (<0.001)
	**Overall**	153	148.62	10707.5 (0.663)	151.90	145.45	7309.0 (0.593)	154.12	126.71		165.24	170.95	10914 (0.012)

D-4	4.1	150.25	150.69	11062.0 (0.936)	150.97	148.78	7526.0 (0.850)	175.75	135.45		189.46	91.51	49869 (<0.001)
	4.2	151.34	149.87	10921.0 (0.871)	151.76	145.95	7341.5 (0.595)	162.67	115.83		175.76	162.04	25053 (<0.001)
	4.3	145.14	154.54	11720.5 (0.297)	147.39	161.75	8369.0 (0.184)	161.01	123.47		199.64	138.06	28520 (<0.001)
	4.4	150.67	150.37	11007 (0.973)	149.92	152.58	7773.0 (0.806)	160.23	146.80		208.36	96.61	47802 (<0.001)
	**Overall**	148.32	152.15	11311 (0.697)	149.51	154.08	7870.0 (0.699)	171.00	131.03		209.40	96.62	51013 (<0.001)

The study results indicated no significant difference in the mean rankings of individuals’ levels of agreement regarding research activities in radiology and challenges in adapting EBP within radiology departments based on participants’ years of experience. In contrast, a significant difference was found in the mean rankings of individuals’ levels of agreement regarding the current practice of EBP within radiology departments based on participants’ years of experience. Participants with 6 to 10 years of experience tended to show higher mean rankings in their level of agreement compared to the other participants (Table 4). In addition, no significant difference was found between all domains and the participants’ qualifications.

**Table 4:** Comparison for four domains according to the sociodemographic variables: (Years of experience, qualification).

Domains		Years of experience				Qualification					
		1–5	6–10	>10	P-value	Diploma	BSc	MSc	PhD	MD	P-value
		Mean Rank				Mean Rank					
**D-1**	1.1	149.65	155.29	147.47	0.462 (0.794)	116.40	142.80	168.38	187.89	181.13	23021 (<0.001)
	1.2	154.73	150.49	141.41	1593 (0.451)	133.78	147.71	119.06	91.28	178.35	18208 (0.001)
	1.3	150.53	157.38	143.46	1107 (0.575)	125.43	149.16	77.50	126.39	178.27	18455 (0.001)
	1.4	153.88	156.84	136.81	2728 (0.256)	123.30	151.25	93.81	105.5	174.10	15352 (0.004)
	1.5	141.87	175.96	143.26	9170 (0.010)	122.80	152.77	110.12	148.78	161.94	6670 (0.154)
	1.6	164.13	141.85	129.94	9219 (0.010)	156.90	163.80	155.56	180.11	104.37	25780 (<0.001)
	1.7	166.91	120.47	145.61	15432 (<0.001)	137.15	160.38	170.94	111.94	130.92	9216 (0.056)
	1.8	143.29	145.20	171.40	8451 (0.015)	157.57	144.34	144.38	134.72	168.01	6324 (0.176)
	**Overall**	156.49	149.39	138.73	2094 (0.351)	119.93	154.76	116.12	129.67	159.42	6695 (0.153)

**D-2**	2.1	148.56	158.31	146.75	0.947 (0.623)	118.98	148.30	88.12	154.83	178.48	17684 (0.001)
	2.2	156.89	159.95	127.16	8097 (0.017)	100.95	152.39	106.62	183.33	168.76	18829 (0.001)
	2.3	145.25	165.36	146.73	3160 (0.206)	131.65	152.75	129.75	182.17	150.85	3554 (0.470)
	2.4	140.32	157.66	165.15	5310 (0.070)	158.00	147.46	182.38	153.78	151.45	1763 (0.779)
	2.5	138.88	181.84	173.74	14081 (0.001)	136.52	154.86	147.88	137.83	146.42	1766 (0.779)
	**Overall**	140.00	172.36	150.94	7048 (0.029)	118.33	149.88	126.44	164.50	168.17	7835 (0.098)

**D-3**	3.1	148.92	175.85	128.21	12769 (0.002)	101.93	160.02	172.38	99.94	149.68	17627 (0.001)
	3.2	150.19	160.97	140.55	2287 (0.320)	119.18	157.65	109.31	134.22	151.58	8282 (0.082)
	3.3	152.52	135.52	161.34	3734 (0.155)	141.73	150.52	153.50	200.00	147.28	3680 (0.451)
	3.4	145.51	155.26	156.42	1206 (0.547)	151.20	154.47	151.50	146.67	139.11	1734 0.784
	3.5	146.19	157.66	152.51	1042 (0.294)	145.97	155.62	142.12	159.00	137.62	2648 (0.618)
	3.6	143.95	159.90	155.06	2093 (0.351)	167.80	151.81	167.00	128.44	139.75	3352 (0.501)
	**Overall**	146.42	159.67	149.89	1176 (0.555)	129.23	156.99	148.31	124.22	145.45	3959 0.412

**D-4**	4.1	137.86	171.99	155.92	8891 (0.012)	169.07	152.32	114.25	95.11	148.78	7233 (0.124)
	4.2	147.75	166.32	140.38	4403 (0.111)	150.67	154.07	146.88	111.56	145.95	2875 (0.579)
	4.3	142.34	165.15	153.22	4448 (0.108)	135.83	149.24	151.25	143.72	161.75	2582 (0.630)
	4.4	139.42	161.89	162.80	6561 (0.038)	157.57	147.19	174.12	159.94	152.58	1517 (0.824)
	**Overall**	137.38	166.35	162.68	7823 (0.020)	162.65	149.19	137.19	123.33	154.08	1916 (0.751)

### Qualitative findings

The interviews with participants revealed three predominant themes: *“Value of the EBP in radiology”*, *“Barriers to incorporating EBP”* and “*Future directions of the EBP implantation in radiology”*. Several subthemes arose within these three overarching themes as revealed by the interview responses.

### Theme 1: Value of EBP in radiology

While most of the study participants demonstrated a strong grasp of the concept of EBP and acknowledged its importance in improving practice and providing high-quality care, some participants expressed uncertainty about how to accurately derive the value of this process in their daily practice:

“*It really steps up the game for radiology services by finding and fixing mistakes, and then making things even better”*. (P18-Radiologists)

“*As a radiographer*, *I’ve come across the term EBP*. *I’m keen to learn exactly what it means and how it can be meaningful in my work”*. (P20-Radiographer)

### Theme 2: Barriers to incorporating EBP

Study participants reported certain challenges they encounter in establishing EBP. Participants indicated that engaging in EBP to implement an evidence-based optimisation strategy is perceived as an initiative led by radiology professionals themselves. However, they contend that this cannot be effectively carried out without consistent support from higher management.

“*I hold the view that improving our work is a collective duty. Leadership at all levels should play a proactive role in fostering an environment where Evidence-Based Practice (EBP) is a norm. This involves facilitating employee development through training, ensuring they have the necessary resources, and setting aside time specifically for the adoption of EBP”.* (P16-Radiologist)

Of the study participants, 31% of them reported that they are relying on experience, either their own or that of a colleague, rather than research-based evidence, when deciding to modify scanning techniques. They expressed a hesitancy to adopt a recommended change in practice from literature or other sources without radiologist permission or request. This is highlighting a preference for experiential knowledge over research evidence.

“*Truthfully, we often lean on the lessons we’ve received from our university education initially. Yet, in the long run, it’s through our personal experiences and a bit of experimentation in our department that we’ll genuinely figure out the most effective methods for our work”.* (P1-Radiographer)

“*In fact*, *there are practitioners who stick to conventional methods typically rooted in personal experience or anecdotal evidence*, *not research*. *Yet*, *in my view*, *relying solely on experience doesn’t always lead to the best outcomes”*. (P10-Radiologist)

Several radiographers noted a limited level of independence regarding applying changes to their practice. Many believed that obtaining approval from radiology personnel (e.g. radiologist) was necessary before introducing a new imaging technique or accept/reject case. Given that radiologists are accountable for interpreting images, radiographers generally accepted their restricted autonomy in selecting X-ray projections, scanning protocols and triage patients (e.g. rejecting an inappropriate case).

“*We lack the autonomy to independently decide to turn down unsuitable cases, alter scanning protocols, or attempt new scanning techniques derived from recent scientific studies. Generally, radiologists hesitate to overlap roles and tend to maintain strict oversight over the duties of radiographers”*. (P5-Radiographer)

“*If you’re perceived as being lower in your department*, *your input may not be fully considered or acknowledged*. *We adhere closely to protocols established by application specialists or/and radiologists*, *and we abide by them”*. (P11-Radiographer)

However, individuals possessing higher qualifications (postgraduate degrees) exhibited motivation and confidence in improving clinical decision-making by integrating the best evidence and patient values with clinical expertise:

“*It falls within my responsibilities to explore novel approaches and incorporate them into practice. I guess it’s up to us to take on this responsibility”.* (P17-Radiographer)

### Theme 3: Future directions of the EBP implantation in radiology

Participants highlighted the necessity for increased training sessions to foster an EBP and research culture among radiology professionals:

*“It would be advisable to conduct regular training sessions on this matter because currently, there is no training available on how to conduct research and impalement EBP”*. (P3-Radiographer)

A supportive organizational culture was also reported by participants as an important strategy to improve EBP within radiology departments:

“*Strong leadership (higher management) support is essential for adapting EBP. Leaders who prioritize EBP can provide resources, training, and encouragement for research, motivating employees to adapt EBP in their daily routines”.* (P15-Radiologist)

Another participant reported the need to comprehend the barriers that might hinder staff from embracing EBP:

“*I believe that the initial step in encouraging EBP within any organization is to understand the barriers that may prevent radiographers or/and radiologists from adapting it”.* (P6-Radiologist)

Study participants also emphasized the significance of enhancing EBP within the radiology curriculum:

“*During my time as a student, the concept of EBP was not part of the curriculum. Therefore, I think it would be advantageous to incorporate it into the educational programme for students”.* (P12-Radiographer)

## Discussion

EBP integrates research principles, clinical history, professional expertise, and patient preferences to inform healthcare decision-making and enhance patients’ outcomes [22]. Failing to incorporate research evidence into clinical practice can result in suboptimal care and poor health outcomes [23]. This is particularly concerning in radiology because of its rapid evolution, including the integration of artificial intelligence, and the numerous risk factors such as radiation exposure, strong magnetic fields, and the use of contrast media [24]. Additionally, neglecting EBP has significant economic implications for the healthcare system [24]. The aim of this study was to highlight the current practice of EBP among radiology professionals including radiologists and radiographers in Saudi Arabia and identify challenges to recommend appropriate targeted interventions.

Several studies have indicated that the research component is not typically regarded as a mandatory requirement within the roles and responsibilities of radiology professionals [25–27].

In this study, the results revealed that most of the study participants strongly agreed on the importance of scientific research in advancing the radiology profession and guiding clinical decision-making. Over half of the participants felt confident in their ability to conduct scientific research in radiology and believed that they should actively initiate projects. Nearly 64% of the participants reported engaging in research activities, and 57% of them were familiar with the research strategies at their workplace. The results showed that individuals who have previously been involved in research and were familiar with workplace research strategies tended to support and advocate for research and EBP more than others. Although the findings highlighted diverse opinions and attitudes regarding who should initiate and lead research activities in radiology, 69% of the participants emphasized the importance of collaboration between academic institutions and healthcare organizations in conducting research in radiology. Similar results were reported by Saukko et al. [21]. This shows the efforts of Saudi Ministry of Health’s (MOH) to encourage their staff members to engage in research by offering financial rewards for their publications. This initiative is part of a broader strategy to promote research excellence and inspire healthcare professionals to advance medical knowledge. The MOH’s dedication to research is in line with Saudi Arabia’s Vision 2030, which emphasises innovation and the enhancement of healthcare services [28]. Research translation centres and academic health science networks in several countries, such as the United Kingdome and Australia have successfully integrated research with healthcare services [25, 29], and such initiatives should be considered in KSA in order to emphasize the importance of these collaborations in fostering innovation and EBP.

The majority of study participants expressed strong agreement regarding their understanding of and familiarity with EBP in radiology. In particular, radiologists demonstrated higher agreement than radiographers concerning their familiarity with EBP and their ability to critically evaluate the quality of research. This aligns with findings from a previous study, which showed that a significant portion of radiologists understand and value EBP in improving patient care [14]. They also agreed on the necessity of EBP in radiology practice and expressed confidence in finding and critically reviewing relevant research [14]. Further research indicated that radiographers struggled with critically evaluating the quality of research to support EBP [14]. Halkett et al. claimed that radiographers often lack confidence in engaging in research and implementing EBP due to insufficient education and expertise in research methodologies [30]. This raises a critical question about whether radiologists receive more extensive training in research methodologies and critical appraisal of scientific literature during their academic preparation compared to radiographers. It suggests that radiographers have less exposure to research training and fewer opportunities for professional development throughout their careers compared to radiologists to implement EBP in their daily practice [8].

Although many participants in this study expressed a high level of agreement regarding their understanding and familiarity with EBP, they reported lower agreement concerning their ability to develop their current practice based on EBP and engage in discussions with colleagues about research evidence. This may be attributed to several reasons, such as lack of training, time constraints, limited access to resources, organisational culture and lack of confidence and program curriculum, all of which can significantly hinder the ability of radiology professionals to develop and enhance their practice based on the latest evidence [31, 32]. This was in line with findings from an earlier study, which found that only 19% reported that they base their current practices on research evidence [21]. In contrast, Saukko et al. found that radiographers showed a preference for discussing research evidence with their colleagues and other professionals [21]. This highlights the importance of integrating EBP into educational programs to help students understand how to critically appraise research, apply findings to clinical practice, and engage in continuous learning [11].

The primary barriers identified by study participants as obstacles to implementing EBP in radiology departments were a lack of knowledge and research skills, insufficient autonomy or authority to implement changes, inadequate support, and preference for experimental knowledge over EBP. Typical responses by study participants included, *“cannot be carried out without consistent support from manager”*, *“lack the autonym to independently decide to make change”*, *and “stick to conventional methods typically rooted in personal experience”*. Numerous studies in the literature have indicated that lack of education, training, and support from managers negatively impacts the implementation of EBP in daily practice [33, 34]. It was noted in this study that those working in private hospitals may struggle more with implementing EBP in their daily work. This may stem from the organisational culture in private hospitals, which might not always prioritise the adaption of EBP. Private hospitals often prioritise financial sustainability and return on investment, which can influence their approach to implementing EBP [35].

In this study, some participants were demotivated to engage with EBP, as they felt that their efforts to implement changes would not be supported or allowed by their organisation/management. This may raise critical concerns about how insufficient autonomy could negatively impact staff satisfaction, self-steam, patient care, and overall effectiveness of healthcare delivery. Healthcare professionals who feel unable to implement the necessary changes may experience lower job satisfaction, leading to higher turnover rates. Healthcare service delivery may also suffer and be compromised as the latest EBP are not applied, potentially resulting in suboptimal outcomes [36]. This suggests further investigation in future research. In contrast, those with higher qualifications expressed motivation and confidence in enhancing clinical decision-making by integrating the best evidence and patient values with clinical expertise. This may indicate that individuals with higher qualifications often excel in both the theoretical and practical aspects of EBP. Their proven competence builds trust with their management, making it more likely for management to rely on their judgments and decisions when implementing necessary practice changes as required [37].

Furthermore, this study revealed that the place of work significantly influenced participants’ positive attitudes towards EBP. Those working in academic hospitals expressed higher agreement regarding research activities and EBP in radiology compared to those working in other types of hospitals. This was similarly found by Saukko et al. and Elshami et al. [21, 38]. This can be attributed to several factors: academic hospitals typically emphasise continuous learning and research, providing more opportunities for training and development in EBP. These institutions often have better access to resources, such as medical databases, scientific journals, and research facilities, which facilitate a deeper understanding and integration of EBP into clinical practice. Additionally, the culture in academic settings usually promotes collaboration and discussion of research findings, fostering a more supportive environment for adapting EBP. Several studies indicated that healthcare professionals in academic settings are more likely to receive formal education and training in EBP, which enhances their knowledge, skills and their daily practice [14, 39].

In addition, the study participants demonstrated a strong willingness to collaborate with students on research projects to promote EBP. They consider students’ graduation projects as valuable opportunities to advance EBP. Most respondents either strongly agreed or agreed on the importance of encouraging students to engage in EBP initiatives. However, the study participants expressed lower agreement about discussing research evidence with students. This was evident in Saukko et al.’s study, which found that engaging in discussions about research evidence with radiology students appears to be relatively uncommon among radiographers, with only 15% of respondents fully agreeing with this practice [21]. This low engagement may be partly attributed to the lack of confidence or expertise effectively discussing research evidence, time pressures, and heavy workload of daily responsibilities.

Improving the current state of EBP among Saudi radiology professionals requires dedicated effort, as highlighted by the study participants. The results showed that providing comprehensive education and ongoing training on EBP with a focus on research methodologies and critical appraisal skills​ are crucial. Developing a culture that values and supports EBP was also reported as a strategy to adapt EBP among radiology professionals. Leadership should encourage research activities, provide time for EBP activities, and recognise and reward efforts to implement EBP in clinical practice. It was also suggested that EBP be incorporated into the educational programme for students. Introducing students to EBP early in their education helps them to develop critical thinking and research skills from the outset [32].

## Limitations

This study aimed to explore the perceptions of radiology professionals regarding the current application of EBP within radiology departments across Saudi Arabia. Consequently, the findings should be interpreted with caution and may not be generalizable to other countries. Given that the respondent group was predominantly radiographers (n = 235) as opposed to radiologists (n = 65), the comparison of the level of agreement between these two professions should be interpreted with caution. Another limitation of this study was that the interviews were conducted virtually, which could have impacted the transcription and interpretation of the qualitative data. This virtual format might lead to issues such as difficulties in capturing non-verbal cues, and potential misunderstandings or misinterpretations of the participants’ responses. Additionally, a lack of personal interaction can affect the depth and clarity of the data collected. To ensure the reliability and validity of the qualitative data collected, interview transcripts were sent back to the interviewees to confirm the accuracy of their statements and provide an opportunity to clarify any ambiguities.

## Conclusion

The study underscores the importance of EBP in radiology, revealing both the recognition of its value and significant barriers to its implementation in Saudi Arabia. Radiologists show a higher familiarity with EBP compared to radiographers. The findings revealed that radiology professionals in Saudi Arabia, particularly those in academic hospitals, demonstrated a strong understanding and familiarity with EBP. However, key challenges include a lack of training, insufficient support, and limited autonomy, which may hinder its implementation. To enhance EBP adoption, comprehensive education, ongoing training, supportive organisational culture, and early introduction of EBP in educational programmes should be considered.

## Supporting information

S1 Checklist Human participants research checklist. (DOCX)

S1 Data (PDF)
