# Supplementary material for: Mapping the future: The current landscape and future directions of evidence-based practice in Saudi radiology departments
Source: PLoS One. 2025 Feb 4;20(2):e0314332. doi: 10.1371/journal.pone.0314332 (PMC11793823; doi:10.1371/journal.pone.0314332)
Supplement: S1 Data — (PDF) [file pone.0314332.s002.pdf]

Gender:	Place of work:	Years of experience:	Qualification level:
Response	Response	Response	Response
2	4	1	2
2	4	1	2
2	4	1	2
1	4	1	2
2	2	1	2
2	1	2	1
1	1	2	1
2	4	1	2
1	2	1	2
1	4	1	2
2	2	1	2
2	2	1	2
2	1	1	5
2	1	2	5
2	4	1	2
1	2	1	2
1	3	1	3
2	3	1	2
1	4	1	2
2	1	1	2
1	1	2	2
2	1	2	2
1	4	1	5
2	2	1	2
1	2	1	2
1	4	2	2
2	4	1	2
2	2	1	2
2	1	1	2
1	2	1	2
1	1	1	2
1	1	2	2
1	1	2	2
1	1	2	3
1	2	1	2
1	1	2	2
1	1	2	2
1	3	1	2
1	3	1	2
1	3	1	2
1	2	1	2
2	3	3	4
1	3	1	2
1	4	1	2
1	2	1	2
1	2	1	2
1	1	2	2
2	2	1	2

1	4	1	2
2	1	2	2
2	4	1	2
2	4	1	5
2	2	1	2
2	2	1	2
1	1	2	2
2	4	1	2
2	1	3	2
2	4	1	2
2	1	3	2
2	4	1	2
1	4	1	2
2	4	1	2
1	1	2	2
1	4	1	2
1	1	2	2
1	1	2	2
1	4	1	2
1	1	2	2
1	1	2	2
2	4	1	2
2	4	1	2
1	1	1	2
2	3	1	2
2	3	1	2
1	4	1	5
1	4	1	2
1	4	1	2
1	4	1	2
2	2	1	2
2	3	1	2
1	2	1	2
1	1	1	2
2	3	1	2
2	3	1	2
2	4	1	2
2	3	1	2
2	4	1	2
2	4	1	2
2	2	3	1
2	2	3	1
2	2	3	1
2	1	3	1
2	1	3	1
1	1	1	2
2	1	1	2
1	1	3	2
1	1	3	1
2	2	3	1

1	1	3	1
1	1	3	2
1	1	3	4
2	2	3	1
1	1	3	5
1	2	3	4
1	1	2	2
1	1	2	2
1	1	3	3
1	1	3	4
2	2	3	4
2	3	1	5
1	1	2	2
1	3	2	2
2	4	1	2
2	4	1	2
2	4	1	2
2	2	1	2
2	4	1	2
2	4	1	2
2	1	2	2
2	4	1	2
2	2	1	2
2	1	1	2
1	2	1	2
2	1	1	2
2	1	1	2
2	1	1	2
2	1	1	2
1	2	1	2
2	1	1	2
1	2	1	2
2	4	1	2
1	2	1	2
1	4	1	2
2	1	2	5
2	1	2	5
2	2	1	5
2	4	1	5
2	1	2	5
2	1	2	5
2	2	2	5
1	4	1	2
1	4	1	2
2	1	2	2
2	4	1	2
1	1	1	4
2	4	1	5
2	1	1	5
1	1	2	5

1	4	1	2
1	4	1	2
1	4	2	3
2	1	2	2
1	1	1	1
1	1	3	2
1	1	2	2
2	1	3	1
2	1	3	1
2	1	3	1
2	1	1	2
2	1	2	2
2	1	1	2
2	1	2	2
2	1	3	2
1	1	1	5
2	1	1	5
2	1	3	1
2	1	1	2
2	1	2	2
2	1	1	2
1	1	2	2
1	1	3	3
2	1	2	2
2	1	2	5
2	1	3	1
2	1	2	5
2	1	3	5
2	1	3	5
1	1	3	5
1	1	3	5
1	2	1	5
1	2	2	3
2	1	1	5
2	1	1	2
2	1	2	2
1	2	2	1
1	1	3	2
1	4	2	2
1	3	3	5
2	1	1	2
1	2	3	3
1	2	2	5
1	2	1	5
1	2	3	1
1	2	3	1
2	1	2	2
2	2	1	2
2	2	1	2
1	3	1	2

2	2	1	2
2	2	3	5
2	2	1	2
2	2	1	2
2	2	1	2
1	2	3	5
2	2	1	2
1	2	3	1
2	2	1	2
1	2	2	2
2	2	1	2
1	2	2	1
2	4	1	2
2	1	1	2
2	2	1	2
1	3	3	5
1	3	3	4
1	1	1	2
2	3	1	2
1	3	3	4
1	2	1	5
1	3	3	4
2	1	3	2
2	1	2	5
1	1	2	5
2	2	1	2
2	2	1	2
2	3	1	2
2	3	2	3
1	1	2	2
2	1	2	5
1	1	3	2
1	1	3	1
2	3	3	5
1	3	3	5
2	1	2	5
1	1	2	5
2	1	2	5
1	1	3	5
2	1	3	5
1	1	2	5
1	2	1	2
1	1	3	2
1	1	2	2
2	1	3	2
2	1	3	1
2	1	3	1
2	1	1	2
2	1	2	2
2	1	1	2

2	1	2	2
2	1	3	2
2	3	3	5
1	3	3	5
2	3	2	5
1	1	2	5
2	1	2	5
1	1	3	5
2	1	3	5
1	3	2	5
2	1	2	2
2	2	1	2
2	2	1	2
1	3	1	2
2	2	1	2
2	2	3	5
2	2	1	2
2	2	1	2
2	2	1	2
1	2	3	5
2	2	1	2
1	2	3	1
2	2	1	2
1	2	2	2
2	2	1	2
1	3	2	5
2	3	3	5
1	3	3	5
2	3	2	5
1	1	2	5
2	2	1	2
2	2	3	5
2	2	1	2
2	2	1	2
2	2	1	2
2	4	1	2
2	4	1	2
2	4	1	2
1	4	1	2
2	2	1	2
2	1	2	1
1	1	2	1
2	4	1	2
1	2	3	5
1	2	3	5
2	2	3	5
2	2	1	2
2	2	3	5
1	2	3	1
1	2	3	1

2	1	2	2
2	2	1	2

Job description:	Unit you work in:	Have you participated in a research activity?
Response	Response	Response
1	1	2
1	1	2
1	1	2
1	3	1
1	3	2
1	4	2
1	1	2
1	1	2
1	2	1
1	3	1
1	3	2
1	3	2
2	1	1
2	3	1
1	1	1
1	3	1
1	4	1
1	1	2
1	1	1
1	1	1
1	3	1
1	3	2
2	1	1
1	1	1
1	1	1
1	4	2
1	1	1
1	3	2
1	4	1
1	1	2
1	1	1
1	3	1
1	3	1
1	3	1
1	4	1
1	3	1
1	3	1
1	4	1
1	4	1
1	4	1
1	2	1
1	1	1
1	4	1
1	4	2
1	1	2
1	1	2
1	1	2
1	3	1

1	1	1
1	2	1
1	1	2
2	1	1
1	3	1
1	3	1
1	1	1
1	1	1
1	1	2
1	1	1
1	1	2
1	2	2
1	4	2
1	3	1
1	4	1
1	1	2
1	4	1
1	4	1
1	1	1
1	4	1
1	4	1
1	1	1
1	1	2
1	1	2
1	3	1
1	3	1
2	2	2
1	1	2
1	1	2
1	1	2
1	3	1
1	3	1
1	1	2
1	3	2
1	3	1
1	3	1
1	2	1
1	3	1
1	1	1
1	4	2
1	4	1
1	4	1
1	4	1
1	1	2
1	1	2
1	1	1
1	2	2
1	3	2
1	1	2
1	4	1

1	1	2
1	1	2
1	4	1
1	4	1
2	5	1
1	2	1
1	5	2
1	5	2
1	5	1
1	2	1
1	2	1
2	1	1
1	5	2
1	4	1
1	1	2
1	2	2
1	2	2
1	1	2
1	1	2
1	2	2
1	2	1
1	2	2
1	2	1
1	4	2
1	1	1
1	1	2
1	2	1
1	2	1
1	2	1
1	2	1
1	2	1
1	2	1
1	1	2
1	2	1
1	1	2
2	2	1
2	2	1
2	3	1
2	2	2
2	2	1
2	2	1
2	5	2
1	1	2
1	3	1
1	1	1
1	1	1
1	4	1
2	1	1
2	1	1
2	1	1

1	2	1
1	3	1
1	4	1
1	1	1
1	1	1
1	1	1
1	3	1
1	1	2
1	1	2
1	1	2
1	1	2
1	1	1
1	1	1
1	1	1
1	1	1
2	3	1
2	3	1
1	1	1
1	1	1
1	1	1
1	1	1
1	1	2
1	3	1
1	3	2
2	3	2
1	1	2
2	3	1
2	3	1
2	3	1
2	4	2
2	1	1
2	1	1
1	2	1
2	1	1
1	1	1
1	1	1
1	1	2
1	1	2
1	3	1
2	2	1
1	2	1
1	1	2
2	3	1
2	3	1
1	3	2
1	1	1
1	1	2
1	1	2
1	1	1
1	4	1

1	2	1
2	2	1
1	2	1
1	2	1
1	2	1
2	4	1
1	4	2
1	4	2
1	3	2
1	3	2
1	4	2
1	1	2
1	1	2
1	3	2
1	2	1
2	2	1
1	1	1
1	3	1
1	5	1
1	1	1
2	2	1
1	1	1
1	1	2
2	1	1
2	4	1
1	2	1
1	1	2
1	1	2
1	4	1
1	1	1
2	2	1
1	3	2
1	5	2
2	2	1
2	2	1
2	4	1
2	4	1
2	3	1
2	4	1
2	3	1
2	4	1
1	1	1
1	1	1
1	3	1
1	1	2
1	1	2
1	1	2
1	1	2
1	1	1
1	1	1

1	1	1
1	1	1
2	2	1
2	2	1
2	4	1
2	2	1
2	3	1
2	4	1
2	2	1
2	4	1
1	1	2
1	1	2
1	1	1
1	4	1
1	2	1
2	2	1
1	2	1
1	2	1
1	2	1
2	4	1
1	4	2
1	4	2
1	3	2
1	3	2
1	4	2
2	4	1
2	2	1
2	2	1
2	4	1
2	2	1
1	2	1
2	2	1
1	2	1
1	2	1
1	2	1
1	1	2
1	1	2
1	1	2
1	3	1
1	3	2
1	4	2
1	1	2
1	1	2
2	4	1
2	2	1
2	4	1
1	2	1
2	2	1
1	3	2
1	1	2

1	1	2
1	1	2

Are you familiar with your workplace's research strategy
Response
2
2
2
1
2
1
2
2
1
1
2
2
1
1
2
1
1
1
1
2
1
2
1
2
1
2
2
2
2
2
1
1
1
1
1
1
1
1
1
1
1
1
1
1
1
1
2
2

2
1
2
2
2
2
1
1
2
2
2
2
2
1
1
2
1
1
1
1
1
1
2
2
1
1
1
1
2
1
1
1
2
2
1
1
2
1
1
1
1
1
1
1
2
2
2
1
1
1

2
1
1
1
1
1
2
2
1
1
1
1
2
2
2
2
2
1
1
2
1
2
2
1
1
1
1
1
1
1
1
1
1
1
2
1
1
2
1
1
1
1
1
2
2
2
2
2
2
2

2
2
1
2
2
1
1
2
2
1
2
2
2
2
2
1
2
2
2
2
1
1
1
2
2
1
1
2
1
1
1
1
1
1
2
2
2
1
1
1
2
1
2
1
1
1
2
2
1
1

1
1
1
1
1
1
1
2
1
1
2
2
1
2
1
2
1
2
1
1
1
1
2
1
1
1
2
2
1
2
1
2
2
1
1
2
2
1
2
1
2
2
1
1
2
2
1
2
2
2

2
2
1
1
2
2
1
2
1
2
2
2
1
1
1
1
1
1
1
1
1
2
1
1
2
2
1
1
2
2
1
1
1
1
1
2
2
2
1
2
1
2
2
1
1
1
1
1
2
2

Perspectives about research in radiology
Research is required to promote the radiology profession.
2
2
5
5
4
4
4
5
5
5
4
4
5
5
4
5
4
5
5
5
5
4
5
4
5
5
5
5
4
4
5
5
5
5
5
5
5
5
5
5
4
5
5
3
5
5
4
4

5
3
5
5
4
4
4
5
4
5
4
5
4
4
5
4
5
5
5
5
5
3
5
3
5
5
5
4
5
4
5
5
5
5
5
5
4
5
5
4
4
4
4
5
5
5
4
5
5
4

3
3
5
4
4
5
4
4
5
4
5
5
4
5
5
5
5
5
5
5
5
5
5
5
5
3
5
5
5
4
4
4
4
4
3
5
5
5
5
5
5
5
5
5
4
5
5
4
5
5

5
4
5
4
4
4
5
2
2
5
4
4
5
5
5
4
4
4
4
4
5
5
4
4
5
4
5
5
4
4
5
5
5
4
4
4
5
4
5
5
4
5
5
4
5
5
4
5
5
4

5
5
5
5
5
5
4
5
5
5
4
5
5
4
5
5
5
5
4
5
5
5
4
4
5
5
5
5
5
3
3
3
4
5
5
5
5
5
5
5
5
4
4
5
2
2
5
4
4
5

5
5
5
5
5
5
5
5
5
5
4
5
5
4
5
5
5
5
5
5
4
5
5
5
4
5
5
5
5
5
5
5
5
5
5
2
2
5
5
4
4
4
5
5
5
5
5
5
5
5

4
5

Clinical decisions in radiographic practice should be based on research evidence.
4
4
5
4
5
5
4
5
4
4
5
5
5
5
4
5
4
5
5
5
5
3
5
5
3
5
5
5
4
5
4
5
5
4
4
5
5
5
5
5
5
5
5
4
5
5
4
5

4
4
4
5
5
5
4
5
4
5
4
5
4
4
5
4
5
5
4
5
5
3
4
2
5
5
5
5
5
4
5
5
5
5
5
5
4
5
5
3
5
5
5
4
5
5
4
4
5
5

4
4
4
5
5
4
4
4
4
3
5
5
4
5
5
5
5
5
5
5
4
5
5
4
5
4
4
4
4
5
4
5
4
5
5
5
5
5
5
5
5
5
5
5
4
5
4
5
5
5

5
3
5
4
3
5
5
5
5
4
4
4
5
4
5
5
4
4
4
4
5
5
4
2
3
4
4
5
4
5
4
5
5
5
5
4
5
5
5
5
5
4
5
4
3
4
5
5
5
4

5
5
5
5
5
4
4
5
4
3
4
5
5
5
5
5
4
4
4
4
5
4
5
5
4
4
5
5
5
3
4
4
2
5
5
5
5
5
5
5
5
3
5
5
5
5
4
4
4
5

4
5
5
5
5
5
5
5
5
5
5
5
5
4
5
5
5
5
5
4
4
5
4
3
4
5
5
5
5
5
5
5
5
5
5
4
4
5
4
5
5
4
5
4
4
4
5
5
3
4

u as a Radiographers/radiologits are competent to conduct research in the radiology fi
5
5
5
4
3
4
5
5
4
4
3
3
5
5
3
5
5
5
4
5
5
2
5
3
5
5
5
5
4
3
4
5
5
3
3
5
5
5
5
5
3
5
5
4
5
5
3
3

5
3
4
5
3
3
4
4
2
5
2
5
5
4
5
4
5
5
4
5
5
3
4
4
5
5
5
5
5
3
4
5
5
5
5
5
3
5
5
4
3
3
3
3
5
4
3
4
5
3

5
4
3
3
4
4
3
3
3
5
5
5
3
2
4
5
5
4
5
5
3
5
4
3
4
2
5
5
5
4
5
4
5
4
2
5
5
4
5
5
5
4
5
5
5
4
2
2
3
3

5
5
4
5
3
4
5
5
5
4
4
5
4
3
5
4
4
5
5
5
3
5
2
4
3
4
5
5
3
5
3
4
4
4
4
5
4
4
4
5
4
4
4
4
4
4
4
4
5
3

5
4
5
5
5
5
4
4
4
4
4
5
4
4
5
5
4
4
4
4
4
4
4
4
5
4
5
5
2
3
4
4
2
5
5
5
5
5
5
5
5
3
4
5
5
5
4
4
5
4

3
5
5
5
5
5
5
5
5
5
4
4
5
3
5
4
5
5
5
5
4
4
4
4
4
5
5
5
5
5
5
4
5
5
5
5
5
5
4
3
4
5
5
5
5
5
5
4
4
4

You as a Radiographers/radiologitsshould be initiators of radiographic research projects
3
3
5
4
3
3
4
5
3
4
3
3
5
5
2
5
3
5
4
5
5
4
5
3
5
5
5
5
3
3
3
5
5
4
3
5
5
5
5
5
3
5
5
4
5
5
3
3

4
3
4
5
3
3
4
4
4
5
4
5
5
5
5
4
5
5
5
5
5
3
4
2
5
5
5
5
5
3
3
5
4
5
5
5
3
5
5
4
3
3
3
4
5
3
4
5
5
3

3
3
3
3
4
4
3
3
4
2
4
5
3
4
3
5
5
4
5
5
4
5
4
4
4
3
5
5
5
5
3
5
5
5
2
4
4
4
5
4
4
4
5
5
4
4
2
3
4
4

5
2
4
4
5
5
5
2
3
4
4
4
3
2
3
3
4
4
3
4
4
5
2
5
3
4
5
5
4
4
4
4
4
3
3
4
4
4
4
5
3
4
4
4
4
5
3
4
4
5

5
3
4
5
5
4
4
5
3
4
4
5
4
3
5
5
4
4
4
4
4
4
3
3
5
3
5
5
3
4
4
4
2
5
5
5
5
5
5
5
5
5
5
5
2
3
4
4
4
3

2
3
5
5
5
5
5
5
5
5
3
4
4
5
5
3
4
5
5
4
4
5
3
4
4
5
5
5
5
5
5
3
4
5
5
3
3
5
4
3
3
4
5
4
4
4
5
3
4
5

3
4

u as a Radiographers/Radiologits should be in charge of radiographic research projec
2
2
3
4
2
3
3
3
4
4
2
2
5
5
2
5
2
4
3
5
5
4
3
2
5
3
3
5
3
4
3
5
5
3
3
5
5
5
5
5
2
5
5
4
5
5
3
2

4
2
4
3
2
2
4
4
2
3
2
3
3
4
5
4
5
5
4
5
5
3
4
2
5
5
3
4
5
3
4
5
4
5
5
5
3
5
3
4
2
2
2
5
5
3
3
5
5
2

3
4
3
2
5
5
5
5
5
2
3
5
5
4
4
5
5
4
3
5
5
5
4
5
4
1
5
5
5
4
3
4
3
4
1
3
3
4
5
3
3
4
5
5
5
3
5
2
3
3

5
5
3
5
2
5
5
5
2
4
2
5
3
5
3
3
4
5
4
5
4
5
2
2
3
4
5
5
3
5
4
4
4
3
3
5
4
3
3
3
3
4
5
4
4
4
3
4
3
3

5
2
4
5
5
3
4
5
5
3
5
5
3
3
5
5
4
4
4
4
4
4
3
3
5
4
5
5
4
4
4
5
2
5
5
5
5
5
5
5
5
2
5
5
5
2
4
2
5
3

5
3
5
5
5
5
5
5
5
5
3
4
3
3
5
2
4
5
5
3
4
5
5
3
5
5
5
5
5
5
5
2
4
5
5
2
2
3
4
2
3
3
3
3
3
3
5
2
4
4

3
4

Radiographic research projects should be initiated and led by healthcare institutions (e.g. MOH
5
5
5
3
2
2
5
5
2
3
2
2
2
2
5
2
5
5
1
2
5
2
5
2
5
5
5
2
4
4
4
5
5
4
2
5
5
3
3
3
2
2
3
2
3
3
2
2

2
1
2
5
2
2
4
3
2
5
2
5
2
1
5
2
5
5
2
5
5
3
3
5
3
3
5
3
4
5
5
3
5
2
3
3
4
3
5
4
2
2
2
5
2
2
3
4
5
2

3
2
4
2
5
4
4
4
5
2
3
3
4
1
4
5
5
4
5
5
5
5
4
5
4
3
4
4
4
4
4
4
5
4
5
2
2
4
5
2
2
4
5
5
5
1
5
5
4
4

5
5
1
5
4
3
5
5
5
4
4
5
4
3
2
2
5
4
4
5
3
5
5
3
3
5
2
2
4
3
4
4
4
2
2
5
4
4
1
3
2
2
2
1
3
2
2
4
3
2

5
3
4
5
5
2
4
5
4
3
3
5
1
2
5
1
5
4
2
5
4
5
2
2
3
2
3
5
2
3
2
5
2
2
3
2
2
2
2
2
2
4
3
5
5
5
4
4
5
4

3
2
2
3
2
2
2
2
2
2
2
4
3
2
5
3
4
5
5
2
4
5
4
3
3
2
2
3
2
2
5
3
4
5
5
5
5
5
3
2
2
5
5
2
2
2
5
3
3
2

Radiographic research projects should be initiated and led by academic institutions only
5
5
5
2
2
2
3
5
2
2
2
2
2
2
2
2
5
3
2
2
1
2
5
2
3
5
5
2
3
5
2
1
1
4
2
1
1
3
3
3
2
2
3
4
3
3
2
2

2
4
4
5
2
2
5
3
2
5
2
5
5
2
1
2
1
1
2
1
1
3
2
5
3
3
5
3
3
3
4
3
5
2
3
3
4
3
5
4
2
2
2
5
2
3
2
4
3
2

3
2
2
2
5
4
4
4
5
1
3
3
4
1
3
5
5
1
5
5
5
5
1
5
1
3
4
4
4
5
4
5
5
5
5
5
5
1
5
5
5
1
5
5
3
2
5
4
4
4

5
2
2
3
4
4
5
5
2
3
4
3
5
3
5
2
2
3
4
3
5
5
4
3
3
2
1
3
4
3
3
1
1
2
2
3
1
3
2
5
2
5
2
2
3
4
2
1
3
2

5
4
4
5
5
2
4
3
4
4
3
5
2
2
5
1
2
2
2
2
1
2
2
2
3
2
2
5
2
4
2
4
4
4
3
2
2
2
2
2
2
4
4
5
5
2
3
4
3
5

3
5
4
3
2
2
2
2
2
2
2
1
3
2
5
4
4
5
5
2
4
3
4
4
3
2
4
3
2
2
5
4
4
5
5
5
5
5
2
2
2
3
5
2
2
2
5
4
3
4

2
1

ion between educational academic and healthcare institutions in conducting radiography research is i
5
5
5
4
5
4
5
5
5
4
5
5
5
5
5
5
5
5
4
5
5
4
5
5
5
5
5
5
4
3
5
5
5
4
4
5
5
5
5
5
5
4
5
4
5
5
5
5

5
4
4
5
5
5
5
4
5
5
5
5
5
5
5
5
5
5
4
5
5
3
3
4
5
5
5
5
5
5
5
5
5
5
5
5
4
5
5
4
5
5
5
5
5
5
3
5
5
5

4
4
4
5
5
4
5
5
5
5
4
5
5
5
3
5
5
4
5
5
5
5
4
5
4
5
5
5
5
4
3
4
5
4
5
5
5
4
5
5
5
4
5
5
3
4
5
5
5
5

5
5
4
3
2
5
5
5
5
4
4
3
5
5
5
4
3
5
4
3
5
5
5
4
4
4
4
5
4
5
4
4
4
4
4
3
4
5
4
5
4
5
4
5
5
5
4
4
3
5

5
5
3
5
5
5
4
5
3
5
5
5
4
4
5
5
5
4
5
5
4
5
4
4
5
5
5
5
5
4
5
4
5
5
5
5
5
5
5
5
5
2
5
5
5
5
4
4
3
5

5
5
5
5
5
5
5
5
5
5
4
4
3
5
5
5
3
5
5
5
4
5
3
5
5
5
5
5
5
5
5
5
3
5
5
5
5
5
4
5
4
5
5
5
5
5
5
5
5
5

4

K2wledge about evidence-based practice (EBP) in radiology
I am familiar with evidence-based research in my profession and/or specialty
2
2
5
3
3
4
3
5
4
3
3
3
5
5
5
5
2
4
4
5
3
2
5
3
5
5
5
5
3
3
4
3
3
2
4
3
3
4
4
4
3
5
4
4
3
3
4
3

2
5
4
5
3
3
5
4
2
5
2
5
2
3
5
2
5
5
4
5
5
3
3
5
4
4
5
4
4
4
4
4
3
5
4
4
4
4
5
4
3
3
3
3
3
4
4
4
3
3

4
4
4
3
5
4
4
4
3
4
4
4
4
2
3
2
2
4
5
2
3
2
4
3
4
4
5
5
5
4
5
4
5
4
4
4
4
4
5
4
4
4
5
5
5
4
3
5
4
4

4
2
4
5
5
5
4
5
5
4
3
5
4
1
3
4
4
1
3
5
5
5
4
4
4
4
5
3
3
4
3
4
4
4
4
5
4
4
4
4
4
4
3
4
4
3
4
4
5
3

4
5
4
4
4
4
4
3
4
4
4
5
4
4
4
5
4
4
4
4
4
4
4
4
4
4
4
4
5
2
4
5
4
4
5
4
4
4
4
4
4
4
5
5
4
5
5
4
3
5
4

1
3
5
4
4
4
4
4
4
4
4
4
5
3
4
5
4
4
4
4
4
3
4
4
4
4
5
4
4
4
4
5
4
4
4
2
2
5
3
3
4
3
5
4
4
4
4
5
4
3

4
4

evaluate critically the quality of research to provide evidence-based practice in my place of wor
2
2
5
3
3
4
3
5
4
3
3
3
4
4
4
4
2
5
4
4
3
3
5
3
5
5
5
4
3
3
3
3
3
3
4
3
3
4
4
4
3
5
4
4
4
4
2
3

1
5
4
5
3
3
5
4
1
5
1
5
2
4
5
3
5
5
4
5
5
3
4
5
4
4
5
3
4
4
3
4
3
4
4
4
4
4
5
4
3
3
3
3
5
2
3
4
3
3

3
2
4
3
3
3
3
3
3
4
5
4
3
2
4
2
2
4
5
2
3
2
4
3
4
3
5
5
5
5
5
5
5
5
4
4
4
4
5
4
4
4
5
5
4
4
4
4
5
5

2
1
4
4
4
5
5
2
1
4
4
4
4
3
3
4
2
4
3
4
5
5
4
4
4
4
4
2
2
4
3
4
4
4
4
4
4
4
4
5
4
4
2
4
3
3
4
4
4
4

4
4
4
4
4
3
4
3
4
4
3
5
4
4
4
5
4
4
3
4
4
4
4
4
4
4
3
5
2
4
4
4
3
5
4
4
4
4
4
4
4
4
5
5
2
1
4
4
4
4

3
3
5
4
4
4
4
4
4
4
4
4
4
4
4
4
4
4
4
3
4
3
4
4
3
4
5
4
4
4
4
4
4
4
4
2
2
5
3
3
4
3
5
3
3
3
4
4
3
3

I develop my current practice on the grounds of evidence-based research
2
2
1
4
3
4
3
1
4
4
3
3
5
5
3
5
2
5
5
5
5
4
1
3
5
1
1
5
3
3
4
5
5
3
4
5
5
4
4
4
3
5
4
4
4
4
3
3

1
5
4
1
3
3
5
4
1
1
1
1
2
5
5
3
5
5
4
5
5
3
4
5
4
4
1
4
5
4
5
4
2
5
4
4
4
4
1
4
3
3
3
5
3
4
4
5
3
3

3
2
4
3
3
3
4
4
5
4
5
4
4
3
4
2
2
3
1
2
5
2
3
5
3
5
5
5
5
5
4
5
1
5
5
4
4
3
5
4
4
3
5
5
1
5
4
5
3
3

4
3
5
1
4
5
4
2
2
4
4
1
3
3
4
4
3
5
4
1
5
5
4
3
4
4
4
2
2
3
3
3
3
4
4
1
3
4
5
5
4
3
4
4
4
5
4
3
5
4

4
4
3
4
4
4
4
3
4
5
3
5
5
4
4
5
4
4
4
4
3
4
4
4
5
4
3
5
3
3
4
5
4
3
4
4
4
4
4
4
4
4
5
4
2
2
4
4
1
3

3
4
3
4
4
4
4
4
4
4
4
3
5
4
4
4
3
4
4
4
4
3
4
5
3
4
3
4
4
4
4
4
3
4
4
2
2
1
4
3
4
3
1
4
4
4
4
4
4
5

4
3

I discuss research evidence with my colleagues (1/2s)
5
5
1
5
2
4
3
1
4
5
2
2
3
3
1
3
5
5
5
3
5
2
1
2
3
1
1
3
3
4
4
5
5
4
5
5
5
4
4
4
2
5
4
4
4
4
2
2

1
3
4
1
2
2
5
5
1
1
1
1
1
4
5
3
5
5
4
5
5
3
4
5
4
4
1
4
5
4
4
4
2
3
4
4
3
4
1
4
2
2
2
5
5
3
4
5
3
2

4
3
5
2
5
4
3
3
5
4
5
4
3
4
4
2
2
4
1
2
5
2
4
5
4
4
5
5
5
5
3
5
1
5
3
4
4
4
5
4
4
4
5
1
4
5
3
4
2
2

5
5
5
4
4
5
4
5
5
4
4
4
3
2
3
4
4
5
4
4
4
4
4
2
3
5
4
2
2
4
3
4
4
4
4
4
4
5
5
5
4
4
4
4
4
5
4
4
5
5

4
4
3
4
4
5
4
3
4
4
4
5
5
4
4
3
3
4
4
3
4
3
4
4
5
4
3
5
2
4
4
4
4
4
4
4
4
4
4
4
4
4
5
4
5
5
4
4
4
3

2
3
4
4
4
4
4
4
4
4
4
4
5
5
4
4
3
4
4
5
4
3
4
4
4
4
4
4
4
4
4
4
3
4
4
5
5
1
5
2
4
3
1
5
5
5
4
4
4
5

4
4

I discuss research evidence with other professionals
4
4
1
5
4
4
4
1
3
5
4
4
4
4
1
4
4
5
5
4
5
2
1
4
5
1
1
4
2
3
4
5
5
4
5
5
5
4
4
4
4
4
4
4
4
4
2
4

1
3
4
1
4
4
5
4
1
1
1
1
1
4
5
4
5
5
4
5
5
3
4
5
4
4
1
1
5
4
4
4
2
4
4
4
3
4
1
4
4
4
4
5
5
3
4
5
3
4

4
3
5
4
5
3
4
4
5
4
5
4
4
4
4
2
2
3
1
2
5
2
3
5
3
2
5
5
5
4
3
4
1
4
3
4
4
3
5
4
4
3
5
1
5
5
3
1
1
1

2
2
5
5
3
5
5
1
1
3
4
5
3
1
3
4
2
3
3
5
3
5
4
3
5
2
4
2
3
4
3
3
3
4
4
5
3
5
5
5
4
2
4
4
3
5
4
3
5
3

5
4
3
5
5
4
4
3
3
4
4
5
5
4
5
3
3
2
4
3
3
3
4
4
5
3
3
5
2
4
4
3
5
4
4
4
4
4
4
4
4
3
5
5
1
1
3
4
5
3

1
3
4
4
4
4
4
4
4
4
4
3
5
3
5
4
3
5
5
4
4
3
3
4
4
4
4
4
4
4
5
4
3
5
5
4
4
1
5
4
4
4
1
4
4
4
5
4
3
5

4
3

Barriers to implementing EBP in radiology departments
Lack of k2wledge and research skills (e.g., discuss or evaluate the research)
5
5
5
3
3
3
4
5
5
3
3
3
4
4
3
4
5
3
4
4
5
5
5
3
5
5
5
4
3
4
4
5
5
4
3
5
5
4
4
4
3
4
4
4
4
4
5
3

4
2
4
5
3
3
5
4
5
5
5
5
5
4
5
4
5
5
4
5
5
3
3
5
4
4
5
3
5
4
5
4
4
4
4
4
4
4
5
4
3
3
3
5
4
4
4
5
5
3

3
2
4
3
4
5
5
5
5
4
3
4
5
5
4
5
5
4
5
5
5
5
4
5
4
4
5
5
5
4
5
4
5
4
3
4
4
4
5
4
4
4
5
5
4
4
4
5
4
4

5
4
4
4
5
5
5
2
2
4
4
4
5
5
5
3
4
3
4
4
5
5
4
4
5
4
5
4
4
4
3
4
4
3
3
4
4
4
4
5
3
4
4
2
3
4
3
4
2
3

3
4
4
3
3
4
3
3
5
4
4
5
4
3
3
4
2
4
4
2
4
2
3
3
4
5
1
5
4
4
4
4
4
4
4
4
4
4
4
4
4
5
5
5
2
2
4
4
4
5

5
5
4
4
4
4
4
4
4
4
3
4
2
3
3
4
4
3
3
4
3
3
5
4
4
4
4
4
4
4
3
4
4
3
3
5
5
5
3
3
3
4
5
4
4
4
3
4
3
4

3
4

Lack of resources (e.g., access to research)	Lack of teamwork culture
5	5
5	5
5	5
4	4
4	4
4	2
3	5
5	5
5	4
4	4
4	4
4	4
4	4
4	4
3	3
4	4
5	5
3	2
1	3
4	4
5	5
3	2
5	5
4	4
5	5
5	5
5	5
4	4
4	5
4	4
4	3
5	5
5	5
3	3
3	2
5	5
5	5
4	4
4	4
4	4
4	4
2	5
4	4
4	4
5	4
5	4
5	5
4	4

4	5
3	2
4	4
5	5
4	4
4	4
5	4
4	5
5	5
5	5
5	5
5	5
5	5
2	3
3	4
5	3
3	4
3	4
3	2
3	4
3	4
3	3
3	3
5	5
4	4
4	4
5	5
2	2
5	5
3	2
5	5
4	4
5	5
4	4
4	4
4	4
4	4
4	4
5	5
4	4
4	4
4	4
4	4
4	4
4	4
3	4
4	4
5	4
4	3
4	4

4	4
2	2
5	5
4	4
4	4
4	5
4	2
4	2
4	4
4	4
4	4
4	4
4	2
3	2
3	3
5	5
5	5
2	2
5	5
5	5
4	4
5	5
2	2
4	4
2	2
5	4
5	4
5	4
5	4
4	3
5	5
4	3
5	5
4	3
3	3
4	4
4	4
2	2
5	5
4	4
4	4
2	2
4	5
2	5
5	3
1	3
1	4
5	5
4	4
4	4

1	2
1	4
1	3
5	3
3	2
4	5
5	5
1	1
1	1
4	4
3	3
5	3
5	4
4	4
5	5
4	2
5	4
4	2
3	5
5	3
5	4
5	5
4	5
5	3
4	3
4	4
5	3
5	4
4	3
4	3
3	2
2	2
2	2
4	2
4	2
5	3
2	2
2	4
1	3
5	5
4	2
4	5
4	4
2	4
3	3
4	5
4	2
2	2
2	2
4	5

3	3
4	4
4	2
3	3
3	3
2	3
3	3
4	5
5	4
5	2
4	4
5	5
1	3
4	2
3	3
4	5
4	4
2	5
4	4
4	4
2	2
4	4
4	2
4	2
4	4
5	4
4	4
5	5
3	3
4	4
4	3
3	4
3	4
5	4
4	4
4	4
4	4
4	4
4	4
4	4
4	4
3	2
4	5
5	5
1	1
1	1
4	4
3	3
5	3
5	4

4	4
5	5
5	4
4	4
4	4
4	4
4	4
4	4
4	4
4	4
4	2
2	2
2	2
4	5
3	3
4	4
4	2
3	3
3	3
2	3
3	3
4	5
5	4
5	2
4	4
4	4
5	4
4	4
4	4
4	4
3	3
4	4
4	2
3	3
3	3
5	5
5	5
5	5
4	4
4	4
4	2
3	5
5	5
2	3
2	3
2	3
3	3
4	4
3	3
4	5

4	2
2	2

Lack of auto2my/authority to apply change	Lack of support
5	5
5	5
5	5
4	4
4	4
3	3
3	3
5	5
5	5
4	4
4	4
4	4
4	4
4	4
3	4
4	4
5	5
3	4
4	4
4	4
5	5
4	4
5	5
4	4
5	5
5	5
5	5
4	4
4	5
4	4
3	5
5	5
5	5
3	3
3	3
5	5
5	5
4	4
4	4
4	4
4	4
4	5
4	4
4	4
4	5
4	5
5	5
4	4

3	4
2	4
5	4
5	5
4	4
4	4
5	5
4	5
5	5
5	5
5	5
5	5
5	5
4	2
3	3
5	5
3	3
3	3
2	3
3	3
3	3
3	3
3	3
5	5
4	4
4	4
5	5
4	5
5	5
2	3
5	4
4	4
4	5
4	4
4	4
4	4
4	3
4	4
5	5
4	4
4	4
4	4
4	4
4	4
4	4
2	4
4	4
5	5
5	5
4	4

4	4
2	2
5	5
4	4
4	4
4	1
3	4
3	4
4	4
4	4
4	4
4	4
3	4
5	5
3	3
4	5
4	5
4	4
5	5
4	5
4	4
4	5
4	4
4	4
4	4
4	4
4	2
4	2
4	2
5	5
3	2
5	5
5	5
5	5
3	3
4	5
4	5
4	4
5	5
4	5
4	5
4	4
5	4
2	4
5	5
4	4
2	5
5	5
4	4
4	4

3	4
2	5
4	4
5	5
5	2
5	5
5	5
5	5
5	5
3	3
3	3
5	5
4	4
5	5
5	5
3	3
2	5
2	5
5	4
5	5
4	4
5	5
4	4
3	5
2	3
2	4
3	4
3	5
5	5
3	3
2	2
4	4
4	4
3	3
3	3
5	5
4	4
5	5
4	4
4	4
3	3
4	5
4	3
4	4
3	3
5	5
3	3
4	4
2	2
3	4

3	3
4	4
2	2
3	3
3	3
2	2
3	3
5	5
5	5
5	5
4	5
5	5
4	4
3	3
3	3
4	5
4	4
3	4
3	4
4	4
4	4
4	4
3	3
3	3
4	3
5	5
5	4
5	5
4	3
4	4
4	4
4	4
4	4
5	4
4	4
4	4
4	4
4	4
4	4
4	4
4	4
5	2
5	5
5	5
5	5
5	5
3	3
3	3
5	5
4	4

5	5
5	5
5	4
4	4
4	4
4	4
4	4
4	4
4	4
4	4
3	3
4	4
2	2
3	4
3	3
4	4
2	2
3	3
3	3
2	2
3	3
5	5
5	5
5	5
4	5
4	4
5	4
4	4
4	4
4	4
3	3
4	4
2	2
3	3
3	3
5	5
5	5
5	5
4	4
4	4
3	3
3	3
5	5
2	2
2	2
2	2
3	3
4	4
3	3
5	5

3	3
4	4

	Perspectives of 1/2s about students and evidence-based practice (EBF
Insufficient time	I discuss research evidence with students in their clinical practice.
5	5
5	5
1	1
2	4
5	5
2	5
3	3
1	1
5	4
2	4
5	5
5	5
4	4
4	4
4	4
4	4
5	5
3	4
2	3
4	4
5	5
5	2
1	1
5	5
5	3
1	1
1	1
4	4
5	3
4	4
5	4
5	5
5	5
4	4
3	5
5	5
5	5
4	5
4	5
4	5
5	5
4	4
4	5
4	4
5	4
5	4
4	2
5	5

5	3
5	5
4	4
1	1
5	5
5	5
5	5
5	4
5	3
1	1
5	3
1	1
5	1
4	2
4	5
4	3
4	5
4	5
3	4
4	5
4	5
3	3
4	4
5	5
4	5
4	5
1	1
3	3
5	5
2	4
4	4
4	5
5	2
4	4
4	5
4	5
3	4
4	5
1	1
4	4
5	5
5	5
5	5
4	4
4	5
3	4
4	5
4	5
5	4
5	5

4	4
2	4
5	4
5	5
4	5
4	3
3	4
3	4
4	4
3	5
5	3
4	5
3	4
5	3
5	4
3	3
3	3
5	2
1	1
3	3
4	4
3	3
5	2
4	4
5	2
4	5
3	5
3	5
3	5
5	5
5	4
5	5
1	1
5	5
5	4
4	3
4	3
5	2
5	5
4	3
4	3
5	2
5	5
4	2
5	5
2	3
4	1
5	5
3	5
3	5

4	2
3	1
2	3
5	5
5	2
4	5
5	5
5	5
5	5
4	4
3	4
5	5
3	3
4	2
5	3
2	5
2	2
4	5
5	4
5	5
3	5
5	5
3	4
5	4
2	4
4	4
3	5
2	2
5	3
3	4
2	3
5	2
5	2
2	5
2	5
5	5
5	2
3	4
2	3
5	4
2	5
5	4
4	3
5	3
3	3
2	4
2	5
5	2
4	4
3	5

3	4
4	4
2	4
3	4
3	4
2	3
3	4
4	5
4	4
2	5
5	4
5	4
2	3
2	5
3	4
4	3
2	3
2	4
3	4
2	3
5	2
2	3
2	5
2	5
5	3
5	4
3	3
5	5
4	2
5	4
3	3
4	5
4	4
5	4
4	5
4	5
4	5
4	5
4	5
4	5
4	5
5	2
4	5
5	5
5	5
5	5
4	4
3	4
5	5
3	3

4	2
5	3
5	4
4	5
4	5
4	5
4	5
4	5
4	5
4	5
2	5
5	2
4	4
3	5
3	4
4	4
2	4
3	4
3	4
2	3
3	4
4	5
4	4
2	5
5	4
4	5
5	4
4	5
4	5
4	5
3	4
4	4
2	4
3	4
3	4
5	5
5	5
1	1
2	4
5	5
2	5
3	3
1	1
2	3
2	3
2	3
3	4
4	4
3	3
2	4

2	5
5	2

P)
I encourage students to search for evidence-based practice.
5
5
5
4
5
5
4
5
5
4
5
5
4
4
3
4
5
4
5
4
5
2
5
5
5
5
5
4
3
4
3
5
5
5
5
5
5
5
5
5
5
4
5
4
4
4
4
5

4
5
4
5
5
5
5
5
4
5
4
5
3
5
5
4
5
5
4
5
5
3
5
5
5
5
5
5
5
4
3
5
3
4
5
5
4
5
5
4
5
5
5
5
5
4
5
4
4
5

4
2
4
5
5
4
5
5
5
5
3
5
5
4
4
2
2
3
5
2
5
2
3
5
3
5
5
5
5
5
3
5
5
5
4
3
3
3
5
3
3
3
5
5
5
5
5
5
5
5

5
5
5
5
3
5
5
5
5
4
4
5
4
4
4
5
4
5
4
5
5
5
4
4
3
4
5
5
4
4
4
3
3
5
5
5
3
5
5
5
5
4
3
4
3
5
5
3
5
5

4
4
4
4
4
4
4
4
5
3
4
5
5
5
4
4
4
4
4
4
3
4
5
5
4
5
4
5
4
4
4
4
4
4
5
5
5
5
5
5
5
3
5
5
5
5
4
4
5
4

4
4
4
5
5
5
5
5
5
5
5
3
5
5
4
4
4
4
4
4
4
4
5
3
4
5
4
5
5
5
4
4
4
4
4
5
5
5
4
5
5
4
5
4
4
4
4
4
3
5

5
3

m willing to participate in research with students to support evidence-based practi
5
5
5
4
5
5
3
5
5
4
5
5
4
4
3
4
5
5
4
4
5
4
5
5
5
5
5
4
4
4
4
5
5
5
5
5
5
5
5
5
5
5
5
4
4
4
4
5

3
5
4
5
5
5
5
5
4
5
4
5
5
5
5
4
5
5
4
5
5
3
5
5
5
5
5
5
5
4
3
5
5
4
5
5
3
5
5
4
5
5
5
5
5
4
5
5
4
5

4
2
4
5
5
5
2
2
5
5
4
5
2
5
4
2
2
4
5
2
5
2
4
5
4
4
5
5
5
4
3
4
5
4
2
4
4
4
5
4
4
4
5
4
5
4
5
5
5
5

4
2
4
5
2
5
5
5
5
4
4
5
4
3
5
5
4
5
5
5
5
5
5
3
4
4
5
5
4
4
3
4
4
5
5
5
4
5
4
5
5
4
5
4
3
4
5
4
5
5

4
4
3
4
4
4
4
4
5
5
4
5
4
5
4
5
4
4
4
4
4
4
5
5
4
5
5
5
4
4
4
5
4
5
5
5
5
5
5
5
5
2
5
5
5
5
4
4
5
4

3
5
5
5
5
5
5
5
5
5
5
4
5
5
4
4
3
4
4
4
4
4
5
5
4
5
5
5
5
5
4
4
3
4
4
5
5
5
4
5
5
3
5
4
4
4
4
4
3
4

find graduation projects of students are an opportunity to support evidence-based practice.	
	5
	5
	3
	4
	5
	5
	4
	3
	5
	4
	5
	5
	4
	4
	3
	4
	5
	5
	2
	4
	5
	4
	3
	5
	5
	3
	3
	4
	4
	4
	2
	5
	5
	5
	5
	5
	5
	5
	5
	5
	5
	5
	5
	4
	3
	3
	4
	5

5
3
5
3
5
5
5
5
5
3
5
3
5
5
5
5
5
5
4
5
5
3
5
5
5
5
3
4
5
4
5
5
5
4
5
5
2
5
3
4
5
5
5
5
5
4
4
5
4
5

4
4
4
5
5
4
2
2
5
4
4
5
2
5
4
2
2
5
3
2
5
2
5
5
5
1
5
5
5
4
3
4
3
4
4
3
3
5
5
3
3
5
5
2
5
2
5
5
5
5

5
5
2
5
4
5
5
5
5
4
4
5
5
5
5
5
4
5
5
5
5
5
5
3
3
4
4
5
4
4
4
5
5
5
5
5
5
3
2
5
5
5
4
2
3
5
5
5
5
5

4
4
3
4
4
4
4
4
4
5
5
5
2
5
4
5
5
4
4
5
5
5
5
5
3
5
5
5
4
4
4
4
4
5
5
5
5
5
5
5
5
4
5
5
5
5
4
4
5
5

5
5
5
5
5
5
5
5
5
5
5
5
5
5
4
4
3
4
4
4
4
4
4
5
5
5
5
5
5
5
4
4
3
4
4
5
5
3
4
5
5
4
3
4
4
4
4
4
3
5

5

Section A:

Demographic information:

1- **Gender:**

Male Female

2- **Place of work:**

Public hospitals (e.g., MOH)

Semi-public hospitals (e.g., Armed force, National guard, specialist hospital)

Private hospitals

University hospitals

3- **Years of experience (years):**

1-5

6 – 10

>10

4- **Qualification level:**

Diploma

BSc

MSc

PhD

Other

5- **Job description:**

Radiologists

Radiographer

Chief radiographer

Other

6- Unit you work in:

General X-ray Unit

US Unit

CT Unit

MRI Unit

NM Unit

7- Have you been participated in research activity?

Yes

No

8- Are you familiar with your workplaces' research strategy?

Yes

No

Section B:

This section concerns your opinion regarding research activities and evidence-based practice in radiology departments.

Code	Items related to Research	Strongly agree	Agree	Neutral	Disagree	Strongly disagree
**PE 1.0**	**Perspectives about research in radiology**					
PE 1.1	Research is required to promote the radiology profession.					
PE 1.2	Clinical decision in radiographic practice should be based on research evidence.					
PE 1.3	You as a radiographer/radiologist is competent to conduct a research in radiology field.					
PE 1.4	You as a radiographer/Radiologist should be initiators of radiographic research projects.					
PE 1.5	You as a radiographer/radiologist					

	should be in charge of radiographic research projects.					
PE 1.6	Radiographic research projects should be initiated and led by healthcare institutions (e.g. MOH) only.					
PE 1.7	Radiographic research projects should be initiated and led by academic institutions only.					
PE 1.8	Cooperation between educational and academic and healthcare institutions in conducting radiography research is important.					
**K 2.0**	**Knowledge about evidence-based practice (EBP) in radiology**					
K 2.1	I am familiar with evidence-based research in my profession or/and speciality.					
K 2.2	I evaluate critically the quality of research to provide evidence-based practice in my place of work.					
K 2.3	I develop my current practice on the grounds of evidence-based research					
K 2.4	I discuss research evidence with my colleagues (radiographers/radiologists)					
K 2.5	I discuss research evidence with other professionals such as radiologists/radiographers.					
**K 3.0**	**Barriers to implement EBP in radiology departments**					
K 3.1	Lack of knowledge and research skills (e.g., discuss or evaluate the research)					
K 3.2	Lack of resources (e.g., access to research)					
K 3.3	Lack of teamwork					
K 3.4	Lack of autonomy/authority to apply change					
K 3.5	Lack of support					
K 3.6	Insufficient time					
**PE 4.0**	**Perspectives of radiographers/radiologists about students and evidence-based practice (EBP)**					
PE 4.1	I discuss research evidence with students in their clinical practice.					
PE 4.2	I encourage students to search evidence-based practice.					
PE 4.3	I am willing to participate in research with students					

	to support evidence-based practice.					
PE 4.4	I find graduation projects of students is an opportunity to support evidence-based practice.					

Please indicate any suggestion to improve the current status of evidence-based research in radiography if it is required?
